# Pre-contrast MAGiC in treated gliomas: a pilot study of quantitative MRI

**DOI:** 10.1038/s41598-022-24276-5

**Published:** 2022-12-17

**Authors:** Laura Nunez-Gonzalez, Karin A. van Garderen, Marion Smits, Jaap Jaspers, Alejandra Méndez Romero, Dirk H. J. Poot, Juan A. Hernandez-Tamames

**Affiliations:** 1 Radiology and Nuclear Medicine, Erasmus MC - University Medical Center, Rotterdam, The Netherlands; 2 Brain Tumor Center, Erasmus MC Cancer Institute, Rotterdam, The Netherlands; 3 Department of Radiotherapy, Erasmus MC Cancer Institute, Rotterdam, The Netherlands

**Keywords:** Diagnostic markers, Predictive markers, Brain imaging

## Abstract

Quantitative MR imaging is becoming more feasible to be used in clinical work since new approaches have been proposed in order to substantially accelerate the acquisition and due to the possibility of synthetically deriving weighted images from the parametric maps. However, their applicability has to be thoroughly validated in order to be included in clinical practice. In this pilot study, we acquired Magnetic Resonance Image Compilation scans to obtain T1, T2 and PD maps in 14 glioma patients. Abnormal tissue was segmented based on conventional images and using a deep learning segmentation technique to define regions of interest (ROIs). The quantitative T1, T2 and PD values inside ROIs were analyzed using the mean, the standard deviation, the skewness and the kurtosis and compared to the quantitative T1, T2 and PD values found in normal white matter. We found significant differences in pre-contrast T1 and T2 values between abnormal tissue and healthy tissue, as well as between T1w-enhancing and non-enhancing regions. ROC analysis was used to evaluate the potential of quantitative T1 and T2 values for voxel-wise classification of abnormal/normal tissue (AUC = 0.95) and of T1w enhancement/non-enhancement (AUC = 0.85). A cross-validated ROC analysis found high sensitivity (73%) and specificity (73%) with AUCs up to 0.68 on the a priori distinction between abnormal tissue with and without T1w-enhancement. These results suggest that normal tissue, abnormal tissue, and tissue with T1w-enhancement are distinguishable by their pre-contrast quantitative values but further investigation is needed.

## Introduction

Characterization of gliomas of the brain has been approached by novel MRI techniques, such as perfusion, diffusion-tensor imaging, diffusion-weighted imaging, amide proton transfer weighted chemical exchange saturation transfer, and dynamic susceptibility contrast^1–5^. The standard assessment of diffuse gliomas includes images such as T1-weighted (before and after the application of contrast agent), T2w/T2w FLAIR and diffusion-weighted images^6,7^.

Fast quantitative MR imaging has the potential of improving these protocols by reducing scan time and reducing variability due to system imperfections^8–10^. However, the sensitivity of quantitative values to changes in normal and disease-affected tissue must be validated. Some relationships between T1 and T2 values and glioma grade have been found in previous studies^11^. Specifically, there have been attempts at characterizing tumor tissue and predicting enhancement using T1 and T2 maps^12–15^, but these focus only on either quantitative T1^12,13^ or quantitative T2^14,15^ but not both. The long scan time needed for the gold standard quantitative techniques^16–18^ makes their inclusion in clinical protocols difficult.

Recently, several fast multi-parametric quantitative image techniques have been developed, such as Magnetic Resonance Fingerprinting (MRF)^19^, Quantitative Transient-state Imaging (QTI)^9^, and Magnetic Resonance Image Compilation (MAGiC)^10^. All of these are able of acquiring quantitative T1, T2, and proton density (PD) maps of the whole brain in less than 6 min, which facilitates their inclusion in clinical protocols and research studies. MRF has been used to differentiate between common types of gliomas^20^. Depending on the glioma type, some deviation in T1 and T2 values has been found but the results were not conclusive so further investigation is needed.

To our knowledge, MAGiC is the only commercial product for simultaneous multiparametric imaging that has been more extensively used in brain tumor patients recently. Several works compare the feasibility of using synthetic weighted images derived from the quantitative maps for clinical assessment in tumors^21–24^. Also, some studies have applied MAGiC to report quantitative relaxometry analysis of gliomas^25,26^, where the difference between pre and post contrast in R1 maps showed significant contrast enhancement in the perilesional area unlike the conventional T1-weighted scans. For multiple sclerosis, an attempt was made to detect enhancing lesions with MAGiC maps without using contrast agent^27^ but with negative results.

In this pilot study, we scouted the potential of multi-parametric maps obtained with a single MAGiC acquisition before contrast injection to extend previous attempts to characterize and differentiate healthy and abnormal tissue and tissue with and without T1w-enhancement, regardless of the type of originally treated tumor. We also investigated the possibility of using multiparametric maps from MAGiC for predicting T1w contrast enhancement in treated diffuse glioma.

## Materials and methods

### Acquisition

Acquisitions were performed with a 3.0 T GE MR750 system and a 3.0 T GE Signa Premier system (General Electric Medical Systems, Waukesha, WI 53,188 USA). A 16 channel Head and Neck array coil was used. The Institutional Review Board from the ethics committee “Medische Ethische Toetsings Commissie Erasmus MC” (https://www.erasmusmc.nl/nl-nl/pages/metc) approved this study prior to the acquisitions. All the study was performed in accordance with the “Research Involving Subjects Act” (WMO) of The Netherlands and in accordance with relevant guidelines/regulations. An informed consent was obtained from all participants and/or their legal guardians. Research involving human research participants have been performed in accordance with the Declaration of Helsinki.

After giving written informed consent, 14 patients were scanned. See supplementary table S1 for age, sex, diagnosis, WHO grade^28^, treatment, extent of resection, and time since last surgery. All patients had undergone surgery on the tumor before the scan. The majority of patients (10/14) had large residual tumor while an additional two patients had some residual tumor after subtotal resection. Only one patient had gross total resection, with no visible tumor on the directly post-operative MRI scan.

For all patients the imaging protocol followed the recommendations from^6^ consisting of pre-contrast T1-weighted (T1W), T2-weighted (T2W), T2w FLAIR and post-contrast T1-weighted (T1c) scans. Additionally, a MAGiC acquisition was included before the contrast agent injection. This was acquired with TE of 92.24 ms, TR of 4000 ms, FOV of 224 mm, slice thickness of 4 mm and voxel size of 0.875 mm × 0.875 mm × 5 mm. The acquisition time for the whole brain with MAGiC was 5 min and 34 s. MAGiC is a sequence based on a Turbo Spin-Echo sequence obtaining the T2 values from the multiple echoes acquired. In addition, saturation prepulses with different delays are applied to encode the T1 values. Once the T1 and T2 quantitative values are estimated, the PD is obtained from the predicted signal intensity at echo time zero. The sequence is time efficient due to avoiding the waiting times after the saturation pulses by reading previously encoded different slices^10,29^.

### Data preparation/ROIs delimitations

The conventional sequences T1w, T1c, T2w and T2 FLAIR were used to segment tissue abnormalities in all patients using HD-GLIO brain tumor segmentation tool^30–32^; . This tool uses a trained neural network to define two regions of interest (ROIs), one for the non-enhancing T2-weighted hyperintensities (T2h) and other for regions with T1w-enhancement after injecting contrast agent (T1e). These regions (T2h + T1e) were combined into a single region of tissue abnormalities (ABN). These tissue abnormalities—with or without contrast enhancement—are known to consist of a mixture of tumor and treatment-related effects, which cannot reliably be distinguished with conventional imaging.

Additionally, we defined an ROI of 1 cm around the entire region of tissue abnormalities (ABN) as perilesional area (PER)^26^, to exclude possible tumoral cells appearing as normal white matter. Finally, an ROI for normal appearing white matter (nWM) was defined as the white matter segmentation from the T1-weighted image using the software for “Statistical Parametric Mapping” (SPM)^33,34^minus the abnormal tissue (ABN) and perilesional area (PER).

For each patient, the quantitative maps obtained with MAGiC were coregistered to the T1-weighted images using the linear image registration tool FLIRT from FSL^35–37^. Subsequently, the quantitative PD, T1 and T2 values were obtained per patient for all the voxels inside the ABN and reported using normalized histograms (probability density function -PDF-).

### Data analysis

To study the **general** distribution of the quantitative values for each patient, the following statistics were computed for each ROI: mean, standard deviation (SD), Skewness, and Kurtosis for PD, T1 and T2 in the nWM, T2h, T1e and PER.

Furthermore, for each ROI, the average and the 95% confidence interval (CI) of the statistical parameters (mean, standard deviation (SD), Skewness, and Kurtosis) across patients were computed and a signed-rank Wilcoxon test was used to detect significant differences between ROIs.

An **voxel-wise** analysis was performed using the receiver operating characteristic curve (ROC)^38^ for three classification questions: ABN vs. nWM; T1e vs. nWM + T2h); and T1e vs. T2h (only inside ABN). The perilesional area was excluded from the ROC analysis because it could contain tumoral tissue under the appearance of normal tissue^25^. For each question four voxel-wise metrics were considered: T1 values, T2 values, the Euclidian norm of the T1 and T2 values (normT1T2) and the Euclidian norm of the logarithm of T1 and T2 values (normlog). Once an ROC curve was defined, the optimal operating point was calculated as the highest Youden’s index^39^ across the entire ROC curve. Similar ROC analysis was performed including PD values: the Euclidian norm of T1, T2 and PD values and the Euclidian norm of the logarithm of T1, T2 and PD values.

In a second step, the threshold obtained from the ROC analysis was applied to the quantitative maps inside the white-matter-mask to compare the selected regions with the initial segmentation.

To predict **T1w-enhancement** from the pre-contrast quantitative maps, patients were divided in two groups based on the presence or absence of T1e within the ABN volume. The average and the 95% CI of the ROI statistics per patient were computed for each group and a signed-rank Wilcoxon test (using the ROI statistics previously calculated for each patient) was performed to study whether there were significant differences between the two groups or not. A ROC analysis was performed and its optimal operating point was calculated (the highest Youden’s index) for each statistical parameter. To evaluate the validity of the ROC analysis, cross-validation was performed by a leaving-pair-out^40^ for all possible combinations and an AUC was obtained for every set. The threshold associated with the optimal operating point was applied to classify the left-out-pair. The occurrence of being classified as T1-enhancement was calculated for each patient. A single AUC was calculated for each parameter as the average of all the AUCs obtained for each set separately.

## Results

### ROIs characterization

This section tries to characterize the regions of interest segmented by HD-GLIO but using quantitative values from multiparametric mapping.

**Figure 1:**
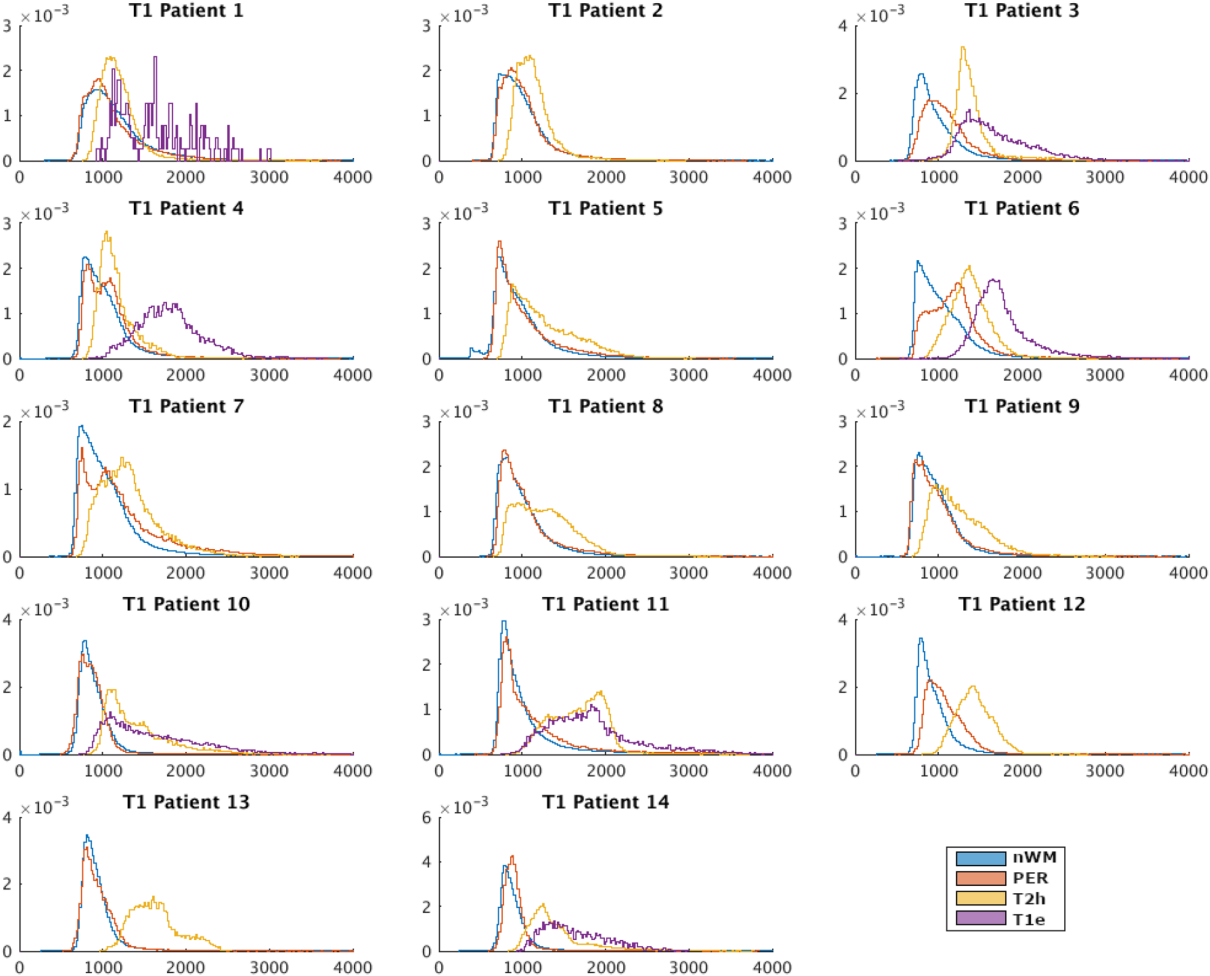
Probability density functions of the T1 (ms) of each patient for each region of interest (Blue→ normal white matter –nWM-, Red→perilesional area –PER-, Yellow→T2 hyperintensity -T2h-, Purple—> T1w-enhancement -T1e-).

**Figure 2:**
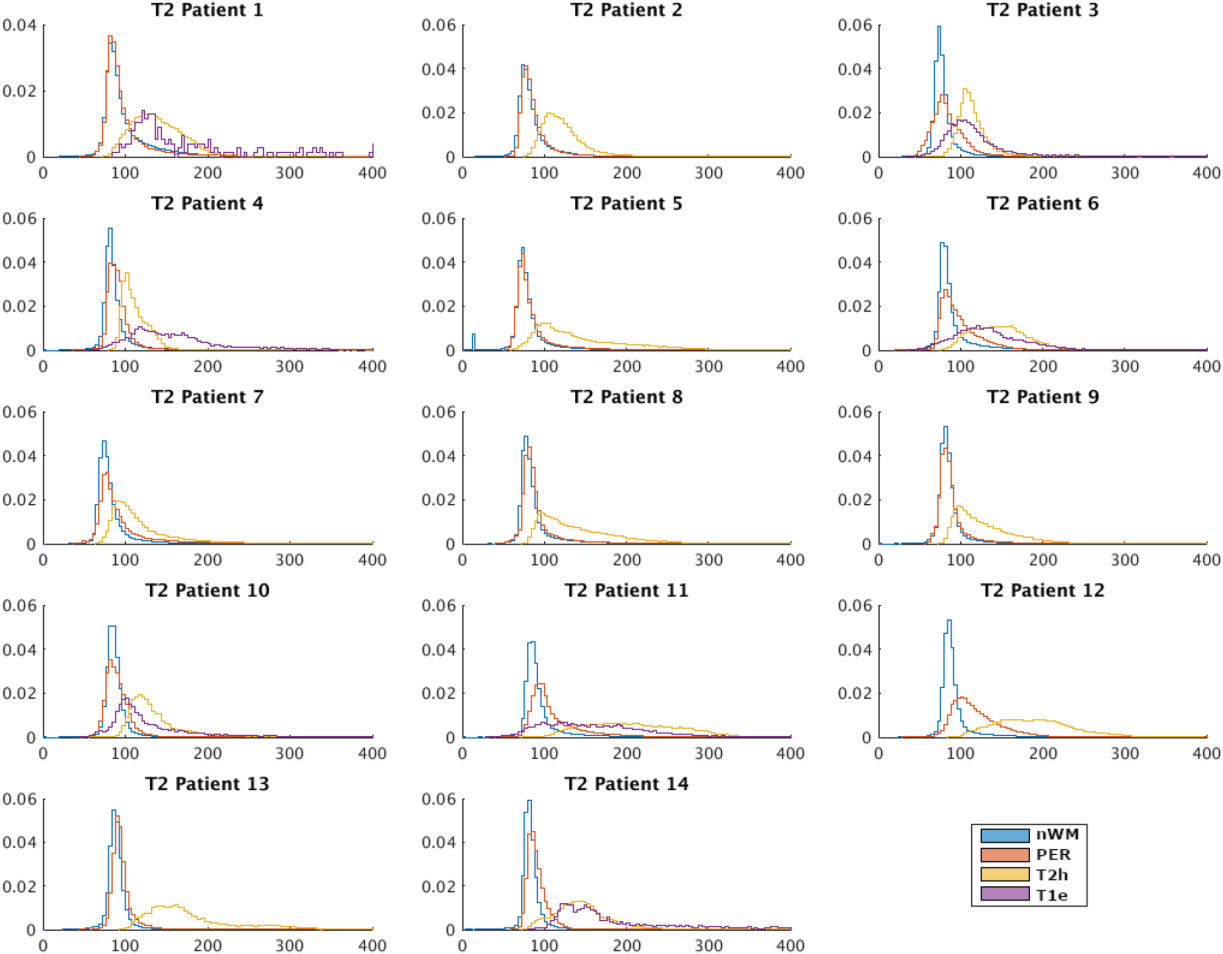
Probability density functions of the T2 (ms) of each patient for each region of interest (Blue→normal white matter –nWM-, Red→perilesional area –PER-, Yellow→T2 hyperintensity -T2h-, Purple—> T1w-enhancement -T1e-).

Figures 1 and 2 respectively show the probability distribution function (PDF) of T1 and T2 of every patient in each of the ROIs. Patients 2, 5, 7, 8, 9, 12 and 13 did not show T1w-enhancement, which is typical for grade 2 diffuse glioma 28. The PDF of PD can be seen in supplementary fig. S1.

Tables 1 and 2 report the average and 95% CI of the ROI statistics of T1 and T2 across all patients as well as the P-values with regards to normal white matter, non-enhanced and enhanced abnormal tissue. The statistics of PD can be seen in supplementary table S2.

**Table 1:** Table with the statistics of the T1 values from all the patients per region of interest (ROI). The ROIs correspond to normal white matter (nWM), T2-hyperintensity (T2h), T1w-enhancement (T1e) and perilesional area (PER). Column 3: Mean over patients of the T1 ROI statistics. Column 4: 95% CI of the mean over patients of the T1 ROI statistics. Column 5: *P*-values of the Wilcoxon signed-rank test with the nWM (P-val ROI vs. nWM). Column 6: *P*-values of the Wilcoxon signed-rank test with the abnormal tissue without T1w-enhancement (P-val ROI vs. T2h). Column 7: *P*-values of the Wilcoxon signed-rank test with the T1w-enhancement voxels (P-val ROI vs. T1e).

ROI	ROI statistic	Mean (ms)	95% CI (ms)	Pval (ROI vs. nWM)	Pval (ROI vs. T2h)	Pval (ROI vs. T1e)
nWM	Mean	993.16	± 38.20	–	< 0.01*	0.02*
	SD	294.04	± 39.61	–	0.95	0.02*
	Skewness	2.64	± 0.57	–	< 0.01*	0.02*
	Kurtosis	16.19	± 6.13	–	< 0.01*	0.02*
T2h	Mean	1353.78	± 92.41	< 0.01*	–	0.02*
	SD	294.68	± 39.78	0.95	–	0.02*
	Skewness	1.23	± 0.39	< 0.01*	–	0.16
	Kurtosis	6.00	± 1.73	< 0.01*	–	0.08
T1e	Mean	1713.30	± 53.19	0.02*	0.02*	–
	SD	448.03	± 51.00	0.02*	0.02*	–
	Skewness	1.00	± 0.19	0.02*	0.16	–
	Kurtosis	4.30	± 0.86	0.02*	0.08	–
PER	Mean	1056.78	± 60.48	0.01*	< 0.01*	0.02*
	SD	315.16	± 65.47	0.24	0.33	0.03*
	Skewness	2.27	± 0.54	< 0.01*	< 0.01*	0.03*
	Kurtosis	13.50	± 6.28	< 0.01*	0.01*	0.03*

*indicates *p* < 0.05.

**Table 2:** Table with the statistics of the T2 values from all the patients per region of interest (ROI). The ROIs correspond to normal white matter (nWM), T2-hyperintensity (T2h), T1w-enhancement (T1e) and perilesional area (PER). Column 3: Mean over patients of the T2 ROI statistics. Column 4: 95% CI of the mean over patients of the T2 ROI statistics. Column 5: P-values of the Wilcoxon signed-rank test with the nWM (P-val ROI vs. nWM). Column 6: P-values of the Wilcoxon signed-rank test with the abnormal tissue without T1w-enhancement (P-val ROI vs. T2h). Column 7: P-values of the Wilcoxon signed-rank test with the T1w-enhancement voxels (P-val ROI vs. T1e).

ROI	ROI statistic	Mean (ms)	95% CI (ms)	Pval (ROI vs. nWM)	Pval (ROI vs. T2h)	Pval (ROI vs. T1e)
nWM	Mean	92.33	± 4.43	–	< 0.01*	0.02*
	SD	48.90	± 10.61	–	0.04*	0.02*
	Skewness	10.72	± 1.75	–	< 0.01*	0.02*
	Kurtosis	184.88	± 57.84	–	< 0.01*	0.02*
T2h	Mean	152.25	± 16.06	< 0.01*	–	0.30
	SD	64.63	± 12.34	0.04*	–	0.02*
	Skewness	4.62	± 1.13	< 0.01*	–	0.08
	Kurtosis	42.38	± 13.60	< 0.01*	–	0.08
T1e	Mean	180.05	± 22.52	0.02*	0.30	–
	SD	130.81	± 45.67	0.02*	0.02*	–
	Skewness	3.57	± 0.95	0.02*	0.08	–
	Kurtosis	24.92	± 11.51	0.02*	0.08	–
PER	Mean	103.82	± 9.75	< 0.01*	< 0.01*	0.02*
	SD	63.56	± 24.05	0.01*	0.50	0.16
	Skewness	8.10	± 1.99	< 0.01*	< 0.01*	0.03*
	Kurtosis	111.55	± 54.74	< 0.01*	< 0.01*	0.03*

* indicates *p* < 0.05.

Table 1 shows that the T1e has the highest T1 followed by T2h, PER and nWM. Also the standard deviation was the highest in T1e followed by PER, and very similar for T2h and nWM. Skewness and Kurtosis were both positive in all the cases (mean Skewness ± 95% CI was nWM = 2.64 ± 0.98, T2h = 1.23 ± 0.68, T1e = 1.00 ± 0.34 and PER = 2.27 ± 0.94 and mean Kurtosis ± 95% CI was nWM = 16.19 ± 10.62, T2h = 6.00 ± 3.01, T1e = 4.30 ± 1.49 and PER = 13.50 ± 10.88), indicating that all the distributions were skewed to the left and had heavier tails than a normal distribution. These values were smaller for T1e than for T2h, PER and nWM had the highest values, which indicate that the distribution of T1 values were closer to a normal distribution for the voxels with T1w-enhancement (ROI T1e). We can see this effect also in Fig. 1.

Using the Wilcoxon test, all T2h, T1e and PER ROI statistics were significantly different from normal white matter (Table 1, column 5), except the SD-T2h (p-value 0.95) and the SD-PER (p-value 0.24). The T1 values in the perilesional area differed from the abnormal tissue in all the parameters except the SD (p-value 0.33) (this was consistent with the no rejection of the hypothesis for SD of nWM). The T1w- enhanced voxels had higher mean than non-enhanced abnormal tissue (Mean difference = 359.52 ms), but other ROI statistics were not significantly different (p-values 0.16 and 0.08 for Skewness and Kurtosis, respectively).

Regarding T2 values, Table 2 shows that T1e had the highest T2, followed by T2h, then PER and, finally, nWM. The standard deviation was also higher for T1e than for the rest. The Skewness and the Kurtosis followed the same trend as T1, indicating distributions more skewed to the left and with higher tails for nWM than PER, T2h and, finally, T1e. Using the Wilcoxon test, the parameters of the distribution of T2 values for T2h, T1e and PER were significantly different (*p*-value < 0.05) from nWM. Also, the parameters for T2 values of PER were significantly different from T2h, except the SD (*p*-value 0.5). However, in contrast to the T1 values, the T2 values of T1e only significantly differed from T2h in SD.

Supplementary table S2 reports similar mean values in PD for the T2h and T1e, although higher than for nWM or the perilesional area. We observed differences in the SD of the perilesional area compared to nWM (*p*-value 0.02) or T2h (*p*-value 0.02) but not compared to T1e (*p*-value 0.94). The Skewness (zero value in the range mean ± SD) and the Kurtosis (positive values) in all the ROIs reflected that the distribution of PD values is symmetric and more tailored than a normal distribution.

#### Voxel-wise characterization

This section tries to obtain an alternative to HD-GLIO segmentation but based on a voxel-wise classification using quantitative parametric maps.

**Figure 3:**
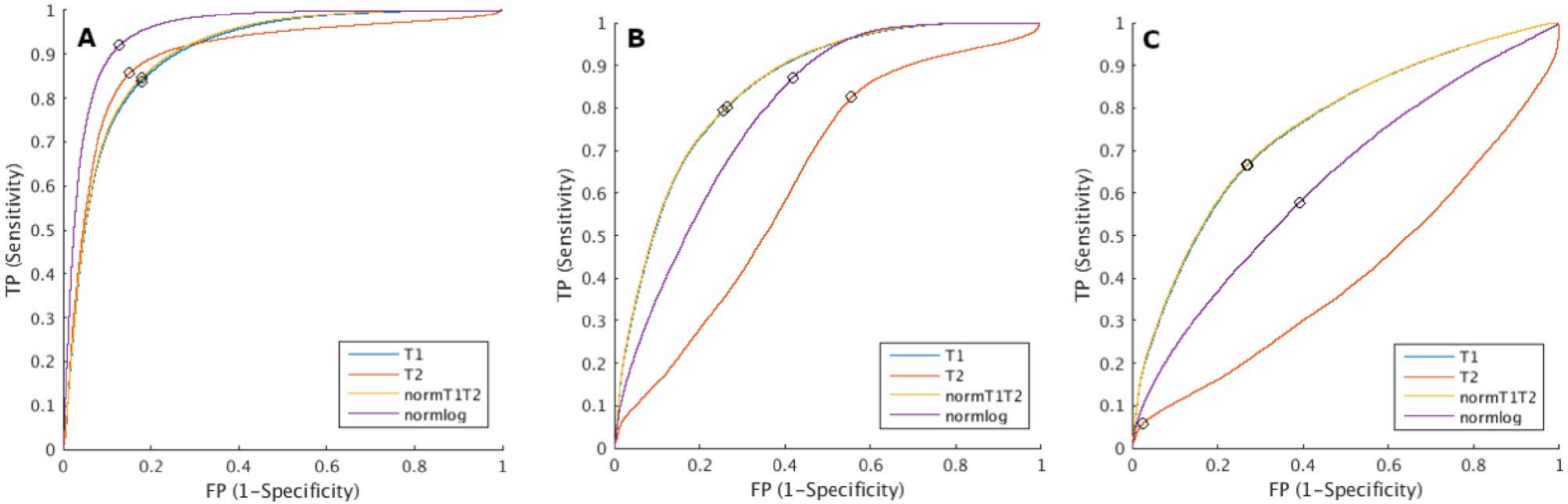
Receiver operating characteristic (ROC) curves using only T1 (blue), only T2 (red), the Euclidian norm of T1 and T2 –normT1T2- (yellow) and the Euclidian norm of the logarithm of T1 and T2 –normlog- (purple). (**a**) ROC curves between the abnormal tissue (ABN) and normal white matter (nWM), (**b**) ROC curves between the T1w-enhanced region (T1e) and the rest of the voxels (T2 hyperintensity region -T2h −  + perilesional area -PER −  + nWM), and (**c**) ROC curves between T1e and T2h. The point with the highest Youden’s index is marked with a black circle.

Figure 3 shows the ROC curves for each of the three questions and all four metrics. To discriminate between ABN and nWM, normlog(Fig. 3A) was the metric with the highest AUC with an value of 0.95. The optimal point (Youden’s index) of the ROC had a sensitivity of 92.03% and specificity of 86.88% at a threshold of 8.44.

For distinguishing T1w-enhancement from the voxels without T1w-enhancement (T2h + PER + nWM), the ROC with highest AUC was normT1T2, shown in Fig. 3B. The AUC was 0.85. Youden’s point had a sensitivity of 81.79% and specificity of 71.99% at a threshold of 1344 ms. Figure 3C shows the ROC analysis distinguishing T1e from T2h within the ABN. In this case, the AUC over the normT1T2 was 0.76 with at Youden’s point a sensitivity of 67.39% and a specificity of 72.26% with a threshold at 1512 ms.

The ROC analysis including PD did not improve the AUC in any of the cases. To distinguish abnormal from healthy tissue (ABN vs. nWM), the highest AUC obtained was 0.95 using the Euclidian norm of the logarithm of T1, T2 and PD. To distinguish T1w-enhancement (T1e), the highest AUCs were using the Euclidian norm of T1, T2 and PD, with an AUC of 0.85 when applying over all ROIs (T2h + PER + nWM) and an AUC of 0.76 when applying vs. T2h only inside the entire region of tissue abnormalities (ABN). The lack of improvement in distinguishing tissue abnormalities and T1w-enhancement motivated the exclusion of PD values in the rest of the analysis.

**Figure 4:**
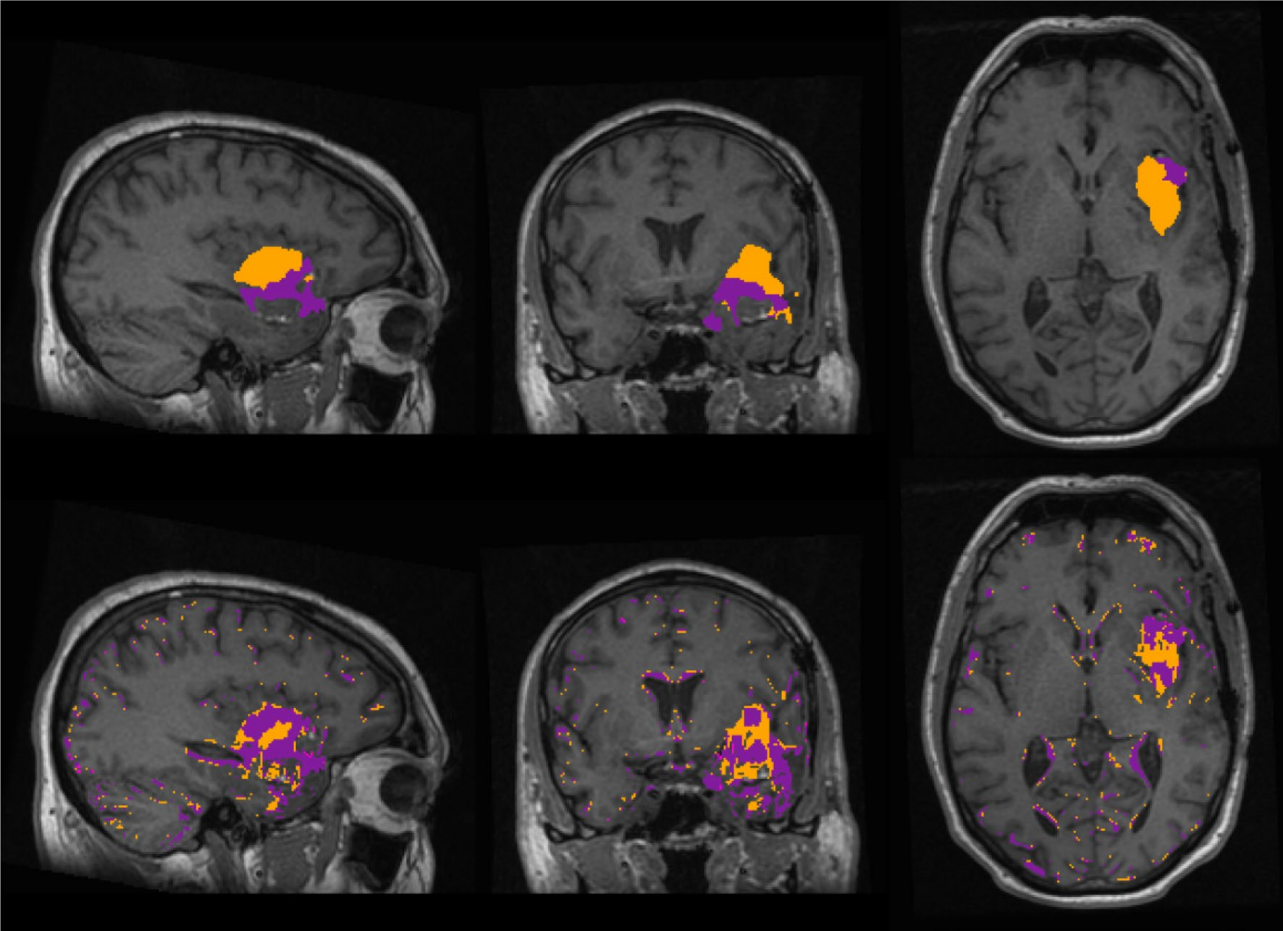
Patient 3. Sagittal, coronal and axial planes of the segmentations overlaid on the T1w scan from. Top: segmentation from HD-GLIO, T2-hyperintensity (T2h) in orange and T1w-enhancement (T1e) in purple. Bottom: using thresholding, voxels classified as abnormal tissue (ABN*) in orange and voxels classified as T1w-enhanced (T1e*) in purple. The purple T1e* region is overlapped with the orange ABN*.

To further inspect the results of the ROC analysis, Fig. 4 shows the segmentations for one representative patient (patient 3) within the white-matter-mask from HD-GLIO and a voxel-wise classification of the T1 and T2 maps. Specifically, using the best ROC results, voxels with normlog > 8.44 were classified as abnormal tissue (ABN*), and voxels with normT1T2 > 1344 ms were classified as T1w-enhancement (T1e*). The segmentations of the other patients are shown in supplementary fig. S2, fig. S3, fig. S4, fig. S5, fig. S6, fig. S7, fig. S8, fig. S9, fig. S10, fig. S11, fig. S12, fig. S13, fig. S14.

In general, by visual inspection there was overlap between the regions segmented by the HD-GLIO tool and the ABN*. The overlap was not perfect, with mismatches at the edges of the region of tissue abnormalities. However, all the ABN* voxels were inside or close to the ABN, or in the limits of the white matter. This effect is easily distinguishable from the ‘lesion’ by the discontinuity and the small size of the patches.

For T1e*, although it captured most of the actual T1w-enhacement in all the patients, it designated regions within the abnormal tissue that are not enhanced as if they were. Even in patients with no T1w-enhancement, T1e* defined big regions inside the abnormal tissue that could be mistaken as T1w-enhancement.

In supplementary table S3, the sensitivity over all the voxels in T1e and ABN, and the specificity and the accuracy over all the voxels in the white-matter-mask after applying the threshold for T1e* and ABN* is reported. In general, the sensitivity was high (> 60%), except in three cases where the sensitivity for ABN* was 55, 59 and 49 for patients 2, 3 and 4 and one case where the sensitivity for T1e* was 59 for patient 10. Mean (95% CI) sensitivity across all the patients was 77 (± 7.5) for T1e* and 69 (± 9.1) for ABN*. The specificity was very high (> = 80%), except in patient 1 with specificity of 78% for T1e* and 68% for ABN*. Mean (95% CI) specificity across all the patients was 89 (± 2.8) for T1e* and 86 (± 4.0) for ABN*. The accuracy was very high (> = 80%), except in patient 1 with accuracy of 78% for T1e* and 69% for ABN*. Mean (95% CI) accuracy across all the patients was 89 (± 2.8) for T1e* and 85 (± 3.8) for ABN*.

### T1w-enhancement prediction

This section of the results is oriented to try to predict voxel enhancement from pre-contrast parametric maps.

**Figure 5:**
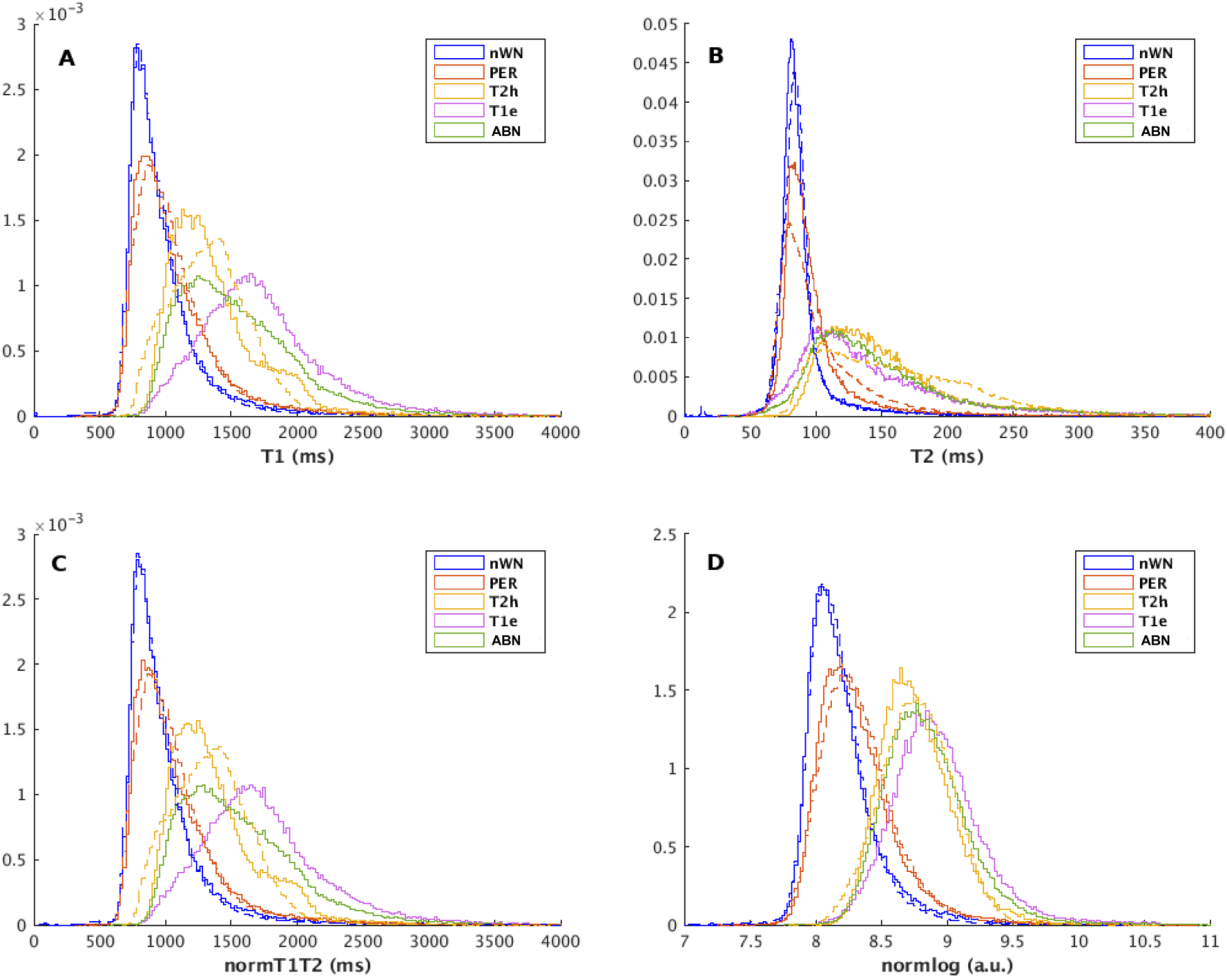
Probability density functions of the T1 values (**a**), T2 values (**b**), the norm of T1 and T2 values –normT1T2- (**c**), and the norm of the logarithm of T1 and T2 values –normlog- (**d**) for all the patients showing T1w-enhancement (solid line) and for all the patients without T1w-enhancement (dashed line) for each region of interest (ROI) (Blue→normal white matter -nWM-, Red→perilesional area –PER-, Yellow→T2w-hyperintensity -T2h-, Purple→T1w-enhancement -T1e-, Green—> Abnormal tissue.

Figure 5 shows the PDF of the T1 values, T2 values, normT1T2, lognorm, for all regions combining all patients with T1w-enhancement and similarly for all patients without T1w-enhancement .

The mean and standard deviation of the statistics (mean, SD, Skewness, and Kurtosis) of T1 and T2 values inside the ABN for each group are reported in Table 3. Also, the P-values of the signed-rank Wilcoxon test between patients with T1w-enhancement and without T1w-enhancement are reported. We observed that although all parameters were higher for the T1w-enhanced regions, only the Skewness and the Kurtosis of T1, and normT1T2 differed significantly.

**Table 3:** Mean and its 95% confidence interval (CI) over subjects in the respective group (T1w-enhancement or no T1w-enhancement) of the region of interest (ROI) statistics of the abnormal tissue for T1 values, T2 values, the Euclidian norm of the T1 and T2 values (normT1T2) and the Euclidian norm of the logarithm of the T1 and T2 values (normlog). The last column gives the P-values of the Wilcoxon signed-rank test between the patient groups.

	Param	Mean No T1w-enhancement	95% CI No T1w-enhancement	Mean T1w-enhancement	95% CI T1w-enhancement	P-val
T1	Mean	1330.20	± 143.34	1457.06	± 139.30	0.08
	SD	299.50	± 58.42	366.34	± 69.23	0.16
	Skewness	0.95	± 0.44	1.51	± 0.35	0.03*
	Kurtosis	4.45	± 1.89	7.02	± 2.46	0.03*
T2	Mean	150.95	± 22.24	157.22	± 25.72	0.47
	SD	61.27	± 7.64	85.71	± 42.36	0.47
	Skewness	3.76	± 1.90	5.05	± 1.19	0.16
	Kurtosis	31.98	± 20.06	45.54	± 20.01	0.22
NormT1T2	Mean	1339.2	± 144.45	1467.13	± 140.11	0.08
	SD	303.98	± 58.70	372.5	± 75.53	0.16
	Skewness	0.99	± 1.93	01.56	± 1.22	0.03*
	Kurtosis	4.69	± 19.93	07.39	± 20.09	0.03*
Normlog	Mean	08.71	± 0.16	08.79	± 0.15	0.22
	SD	0.34	± 0.24	0.35	± 0.36	1.00
	Skewness	0.8	± 0.37	01.2	± 0.27	0.08
	Kurtosis	04.24	± 0.99	06.01	± 0.53	0.05*

*indicates *p* < 0.05.

Table 4 further shows that the highest AUC was obtained using T1 and the normT1T2 as parameter (AUC = 0.68). In both cases, the occurrence was the same for all the patients, and the sensitivity, specificity, and accuracy were 73%, which also was the highest sensitivity and accuracy among all tested statistics. The specificity using the SD of T1 and normT1T2, and the Skewness of normlog was higher than 73% but at the cost of very low sensitivity. The statistic parameters from T2 values were the least accurate, with sensitivities between 29 and 65% and specificity < 50%, except for the SD which did not have false positive values.

**Table 4:** Averaged area under the curve (AUC) of the receiver operating characteristic (ROC) analysis on distinguishing between tumors with and without T1w-enhancement and its standard deviation between brackets from all the leave-pair-out sets used for cross-validation for each statistical parameter. Last three columns report the sensitivity, specificity and accuracy taking into account all the sets and the results showed in Table 4.

	Param	Mean AUC (SD)	Sensitivity	Specificity	Accuracy
T1	Mean	0.62 (0.03)	59%	71%	65%
	SD	0.65 (0.03)	45%	86%	65%
	Skewness	0.65 (0.03)	59%	73%	66%
	**Kurtosis**	**0.68 (0.03)**	**73%**	**73%**	**73%**
T2	Mean	0.54 (0.04)	41%	43%	42%
	SD	0.58 (0.03)	29%	100%	64%
	Skewness	0.58 (0.03)	61%	43%	52%
	Kurtosis	0.56 (0.03)	65%	35%	50%
NormT1T2	Mean	0.62 (0.03)	59%	71%	65%
	SD	0.65 (0.03)	45%	86%	65%
	Skewness	0.66 (0.03)	61%	63%	62%
	**Kurtosis**	**0.68 (0.03)**	**73%**	**73%**	**73%**
Normlog	Mean	0.61 (0.03)	71%	71%	71%
	SD	0.52 (0.04)	73%	31%	52%
	Skewness	0.61 (0.03)	57%	86%	71%
	Kurtosis	0.66 (0.03)	61%	63%	62%

Significant values are in [bold].

Table S4 reports the occurrence of a patient being classified as patient with T1w-enhancement, based on statistics in ABN, during the leave-pair-out cross-validation process with 49 folds in total. Table 4 reports the averaged AUC obtained^40^ as well as the sensitivity, specificity and accuracy from the leave-pair out cross-validation . In all the cases, patient 2 was misclassified as T1w-enhanced when using the Skewness and the Kurtosis as parameter. Using the ‘Mean’ as discriminative parameter, patients 12 and 13 were always misclassified as T1w-enhanced.

## Discussion

The results show that the T1 and T2 values allow partial differentiation of abnormal tissue from normal white matter after surgery across all types of tumor.

While not perfect, it was possible to distinguish voxels with and without T1w-enhancement with normT1T2 with an AUC of 0.85resulting in a mean sensitivity of 77% and a mean specificity of 89% (Table S3) when applying the threshold to each patient separately. Using PD did not increase AUC in any of the cases, hence it was excluded from the voxel-wise experiments and from the T1w-enhancement analysis. However, we suggest that PD should be further investigated as PD showed significant differences between the regions.

We applied voxel-wise thresholds on the quantitative T1 and T2 maps to identify if they contain information about presence of ABN and/or T1e. Reasonable overlap with the HD-GLIO segmentations was observed, though voxel-wise matching was not achieved. However, that is also not expected as HD-GLIO is a segmentation tool that makes use of the T2-weighted FLAIR sequence and is trained to incorporate high-level features and hence spatial context.

By visual inspection of the ROIs of ABN* (those voxels over the threshold defined for abnormal tissue detection), the strong similarity with the lesion segmented by HD-GLIO tool is obvious. Even if they are not perfectly matching, most of the inner lesion is above the threshold with sensitivity ≥ 50 in all. Furthermore, we don’t observe clusters above the threshold in normal white matter. The voxels over the threshold outside the lesion, belong either to the edge of the white matter or are in the perilesional area. The latter is not surprising as the perilesional area may contain tumoral cells or be affected by the tumor (like having a leaky blood brain barrier)^25,41^. The false positives along the edges of the brain could be caused by the partial-volume effect between brain-voxels and CSF which are not properly masked by the white-matter-mask.

Regarding voxel-wise T1w-enhancement, the voxels over the threshold defined a clear cluster in all the patients with T1w-enhancement, although not fully aligned with the original T1e ROI. However, the amount of misclassified voxels without enhancement as T1e* makes the voxel-wise distinction of the T1w-enhancement difficult, since it could easily lead to false positive detection of T1w-enhancement. Although it is possible that it could reflect some leakage in the blood brain barrier not appreciable in conventional images^22,23^, also it could be that only applying a threshold to the pre-contrast quantitative images has moderate ability to detect T1w-enhancement, as it was previously published for Multiple Sclerosis lesions^27^. Further investigation could depict more insights regarding the physiological process underlying the leakage in the blood brain barrier. Another subject of research could be the effects of the treatment in the imaging, and if these effects could be misclassified as a tumor.

Furthermore, the analysis done on abnormal tissue showed that, in almost all the cases, it is possible to discriminate between regions with and without T1w-enhancement with AUCs > 0.6^42^, and providing a sensitivity, specificity and accuracy up to 73%. It is challenging due to the similarities of the quantitative T1 and T2 values of the abnormal tissue with and without T1w-enhancement. Still, it seems that the process of contrast leakage is correlated with the structural information obtained in the pre-contrast scans. The exact mechanisms should be investigated further, but these findings suggest the possibility of detecting blood barrier damage using quantitative images without contrast-agent injection (blood–brain barrier damage is usually measured using corrected cerebral blood volume^5^). This study is limited because of the small number of patients and the use of ROI statistics. Hence, the results encourage further investigation using quantitative imaging to predict T1w-enhancement. Also, intentionally this study excluded the analysis in the same fashion of the conventional weighted images, since we consider the rapid acquisition of MAGiC as a big advance in MRI.

Avoiding contrast agent could mean an improvement for brain tumor patients who need to undergo repeated MRI acquisitions. Some work was previously done using fast quantitative imaging to detect T1w-enhancement. Although successful, in these cases contrast-agent injection was needed^25^. Equally relevant is the reduced scan time (less than 6 min for the whole brain) compared to the 20 to 30 min for a conventional protocol^6,7^. This study is an initial attempt to explore the parametric maps obtained with MAGiC in treated glioma patients. However, to validate these findings more patients should be analyzed. Also, including treatment-naive patients would help identify differences between tumor tissue and treatment-related tissue abnormalities. Furthermore, the finding that pre-contrast quantitative imaging is predictive for the T1w-enhancing region is applicable only to treated gliomas and it may be not the case for other diseases, as other authors showed negative results in detecting T1w-enhancement in patients with Multiple Sclerosis lesions without injecting contrast-agent using quantitative imaging 27. Moreover, distinction between different types and stages of tumors could be reflected by their quantitative values20 and it should be separately investigated.

Classification of voxels in nWM, T1e and T2h could probably be improved, e.g. by taking into account neighboring voxels or more advanced deep learning based classification techniques as also used in the HD-GLIO tool. Also, the possibility of accurately determining the enhancement status of some tumors could prevent the use of contrast-agent in those. Additionally, such probabilistic prediction could be useful in cases where contrast agent is not available or cannot be administered.

In this initial work, we aimed to identify the information present in the individual voxels as ultimately that forms the basis for any such more advanced technique.

## Conclusions

The data analyzed in this work shows there are clear differences in the T1 and T2 quantitative values for the post-treatment tissue abnormalities and healthy tissue. Also, in treated glioma the pre-contrast Euclidian norm of the quantitative T1 and T2 values is predictive for abnormal tissue enhancement . PD was not relevant in this study but it presents different characteristics than the T1 and T2 values, suggesting that more complex analysis could benefit from the quantitative PD values. This pilot study encourages further exploration of quantitative imaging in brain gliomas using MAGiC with the possibility of reducing scan-time and avoiding contrast-agent administration.

## Supplementary Information

Supplementary Information 1.

Supplementary Information 2.

## Data Availability

The datasets used and/or analysed during the current study available from the corresponding author on reasonable request.
