# Supplementary material for: Pre-contrast MAGiC in treated gliomas: a pilot study of quantitative MRI
Source: Sci Rep. 2022 Dec 17;12:21820. doi: 10.1038/s41598-022-24276-5 (PMC9759533; doi:10.1038/s41598-022-24276-5)
Supplement: Supplementary file 1 — Supplementary Information 1. [file 41598_2022_24276_MOESM1_ESM.docx]

Supplementary Images


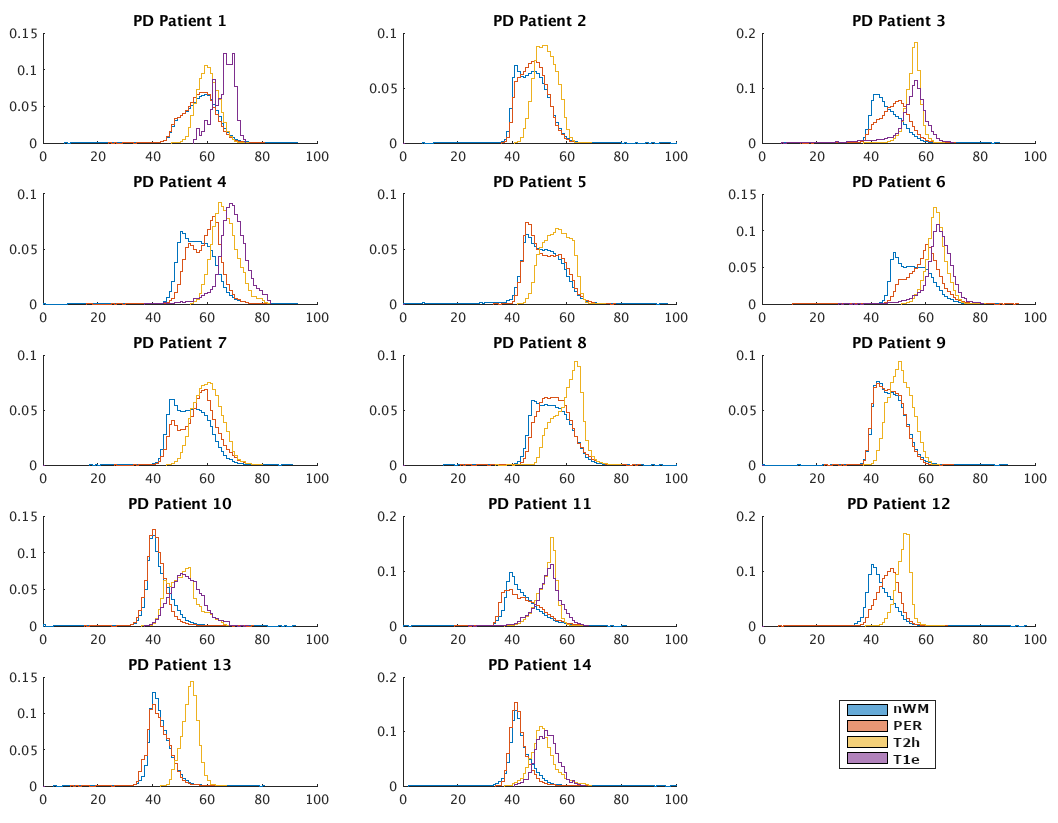


**Figure S1.** Probability density functions of the proton density (PD) (a.u.) of each patient for each region of interest (Blue-> normal white matter –nWM-, Red-> periABNoral area –PER-, Yellow-> T2 hyperintensity -T2h-, Purple -> T1w-enhancement -T1e-)


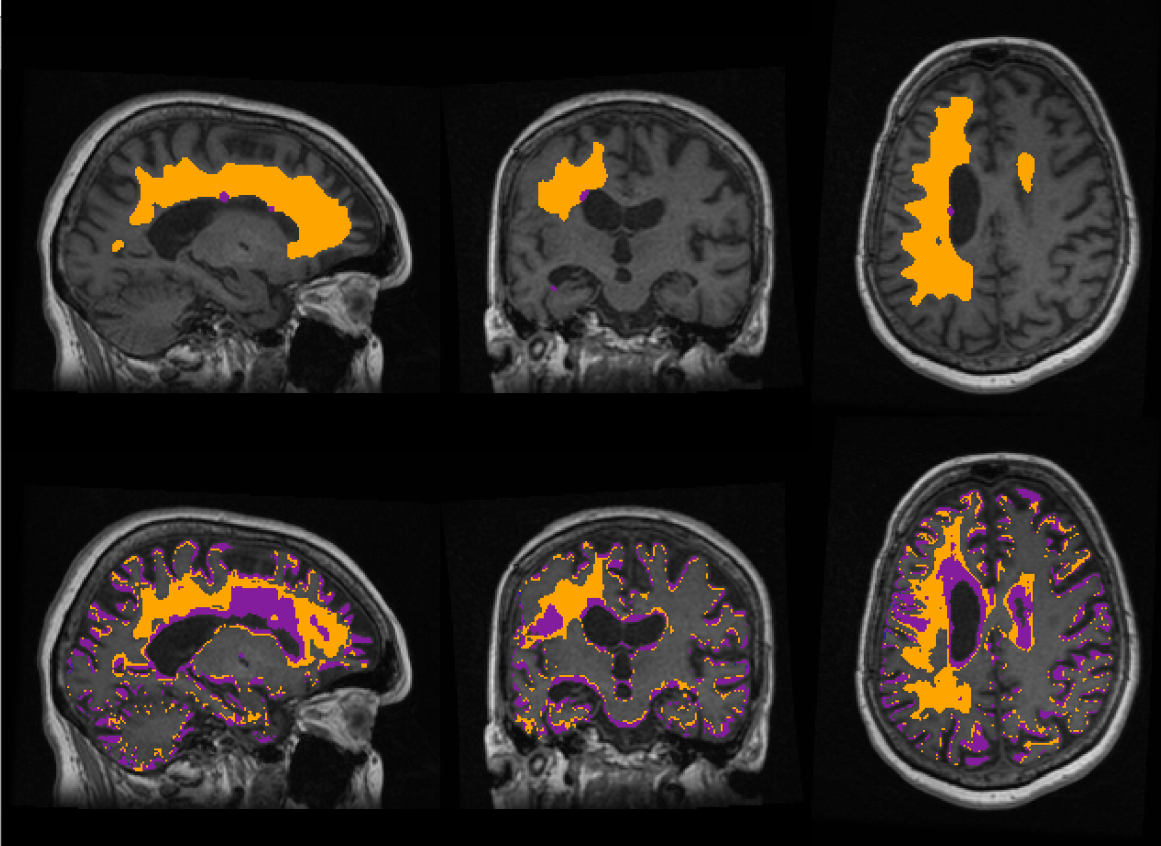


**Figure S2.** Patient 1.Sagittal, coronal and axial planes of the segmentations overlaid on the T1w scan. Top: segmentation from HD-GLIO, T2h in orange and T1e in purple. Bottom: ABN* in orange and T1e* in purple. The purple T1e* region is overlapped with the orange ABN*.


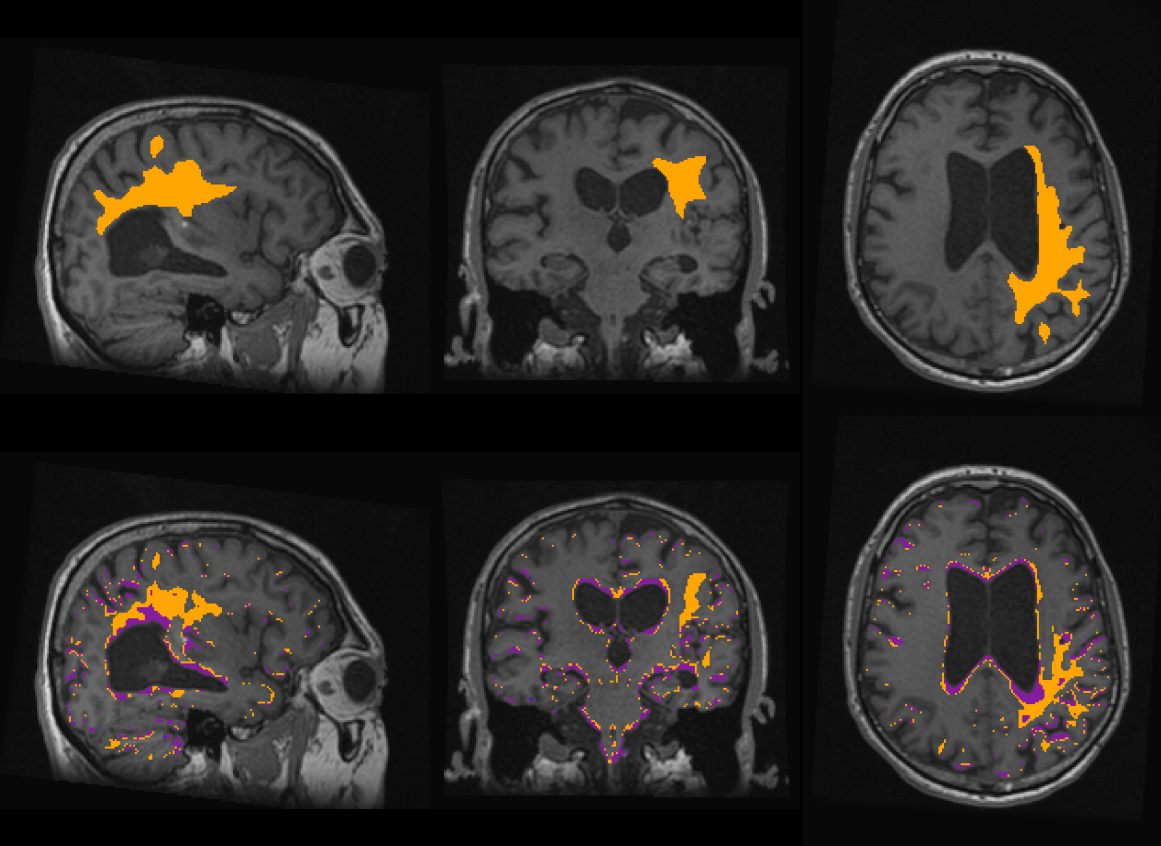


**Figure S3.** Patient 2. Sagittal, coronal and axial planes of the segmentations overlaid on the T1w scan. Top: segmentation from HD-GLIO, T2h in orange, no T1-enhancement. Bottom: ABN* in orange and T1e* in purple. The purple T1e* region is overlapped with the orange ABN*.


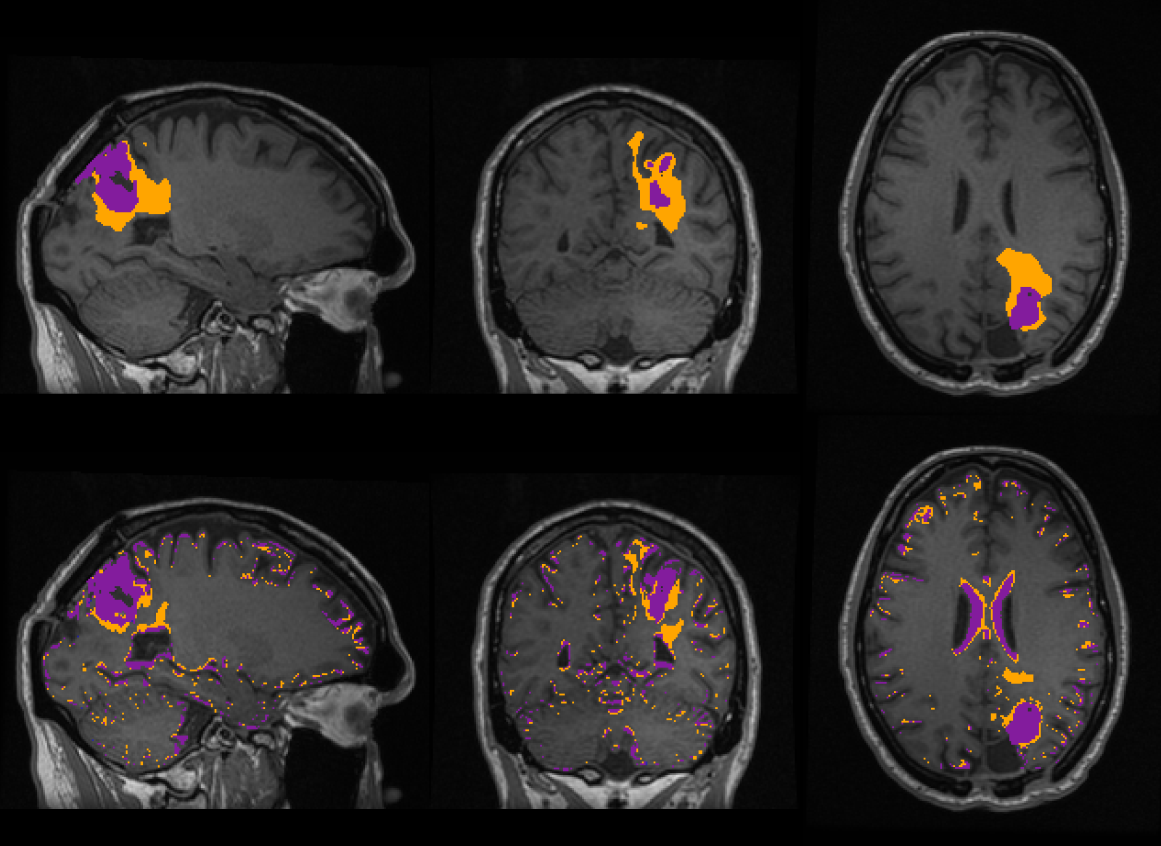


**Figure S4.** Patient 4.Sagittal, coronal and axial planes of the segmentations overlaid on the T1w scan. Top: segmentation from HD-GLIO, T2h in orange and T1e in purple. Bottom: ABN* in orange and T1e* in purple. The purple T1e* region is overlapped with the orange ABN*.


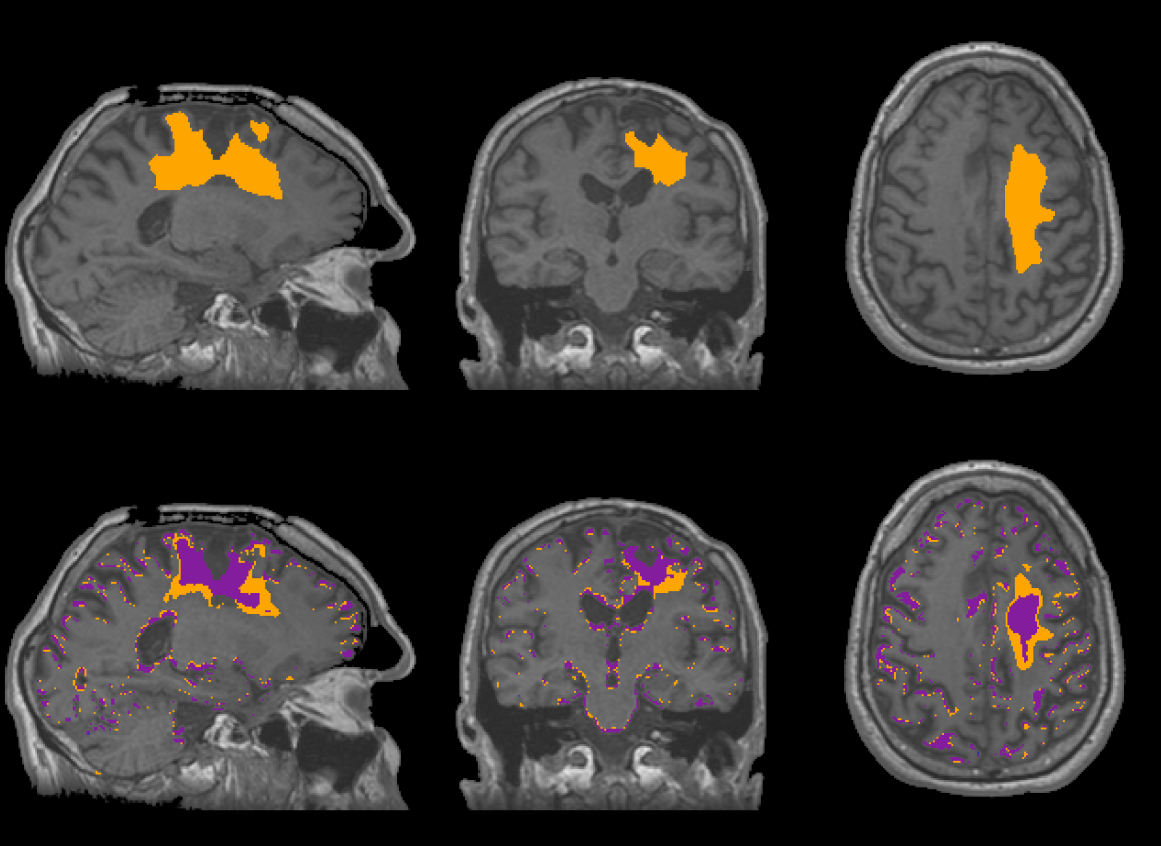


**Figure S5.** Patient 5. Sagittal, coronal and axial planes of the segmentations overlaid on the T1w scan. Top: segmentation from HD-GLIO, T2h in orange, no T1-enhancement. Bottom: ABN* in orange and T1e* in purple. The purple T1e* region is overlapped with the orange ABN*.


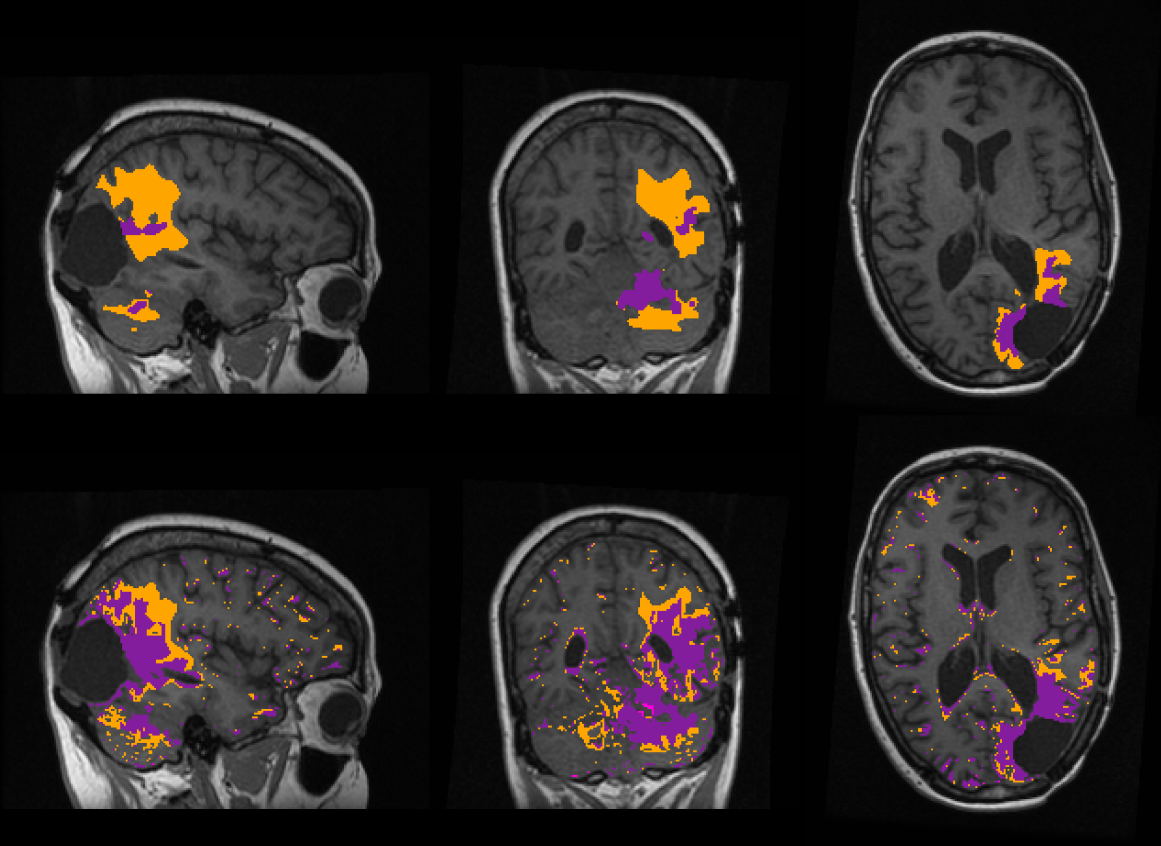


**Figure S6.** Patient 6. Sagittal, coronal and axial planes of the segmentations overlaid on the T1w scan. Top: segmentation from HD-GLIO, T2h in orange and T1e in purple. Bottom: ABN* in orange and T1e* in purple. The purple T1e* region is overlapped with the orange ABN*.


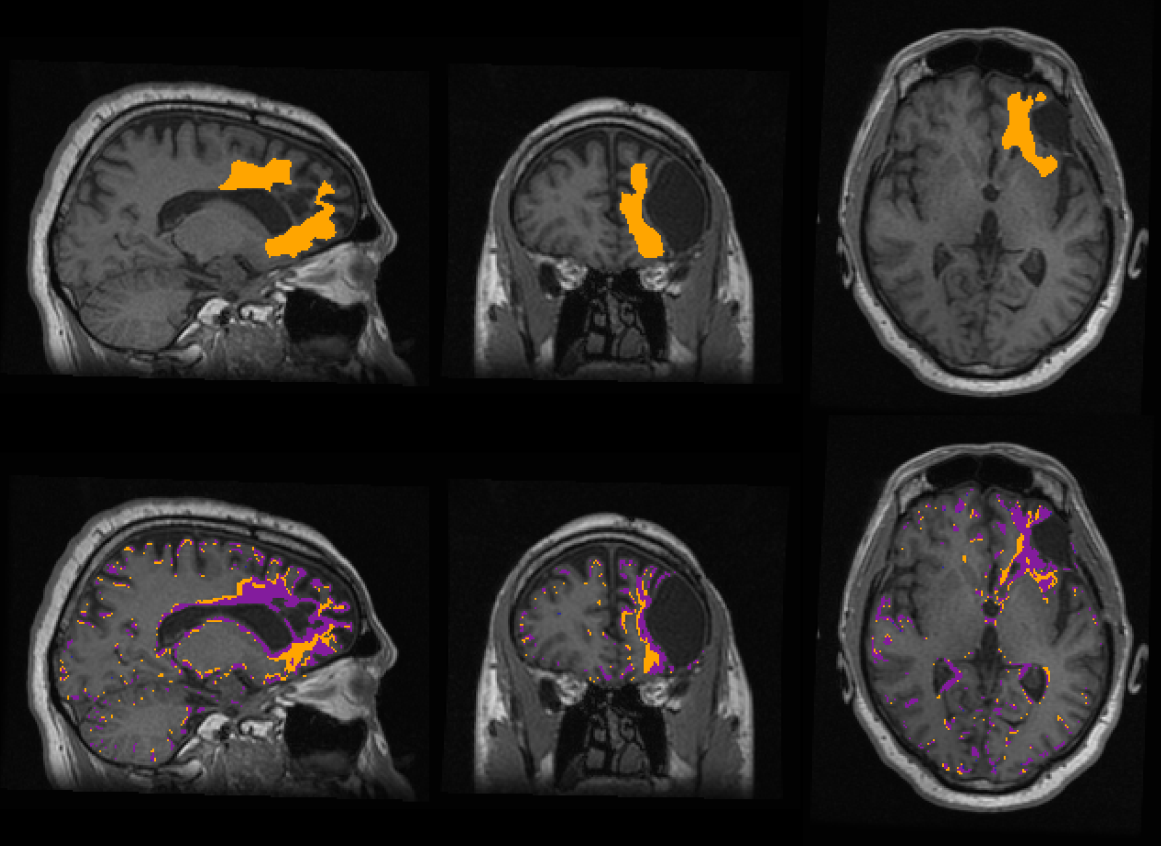


**Figure S7.** Patient 7. Sagittal, coronal and axial planes of the segmentations overlaid on the T1w scan. Top: segmentation from HD-GLIO, T2h in orange, no T1-enhancement. Bottom: ABN* in orange and T1e* in purple. The purple T1e* region is overlapped with the orange ABN*.


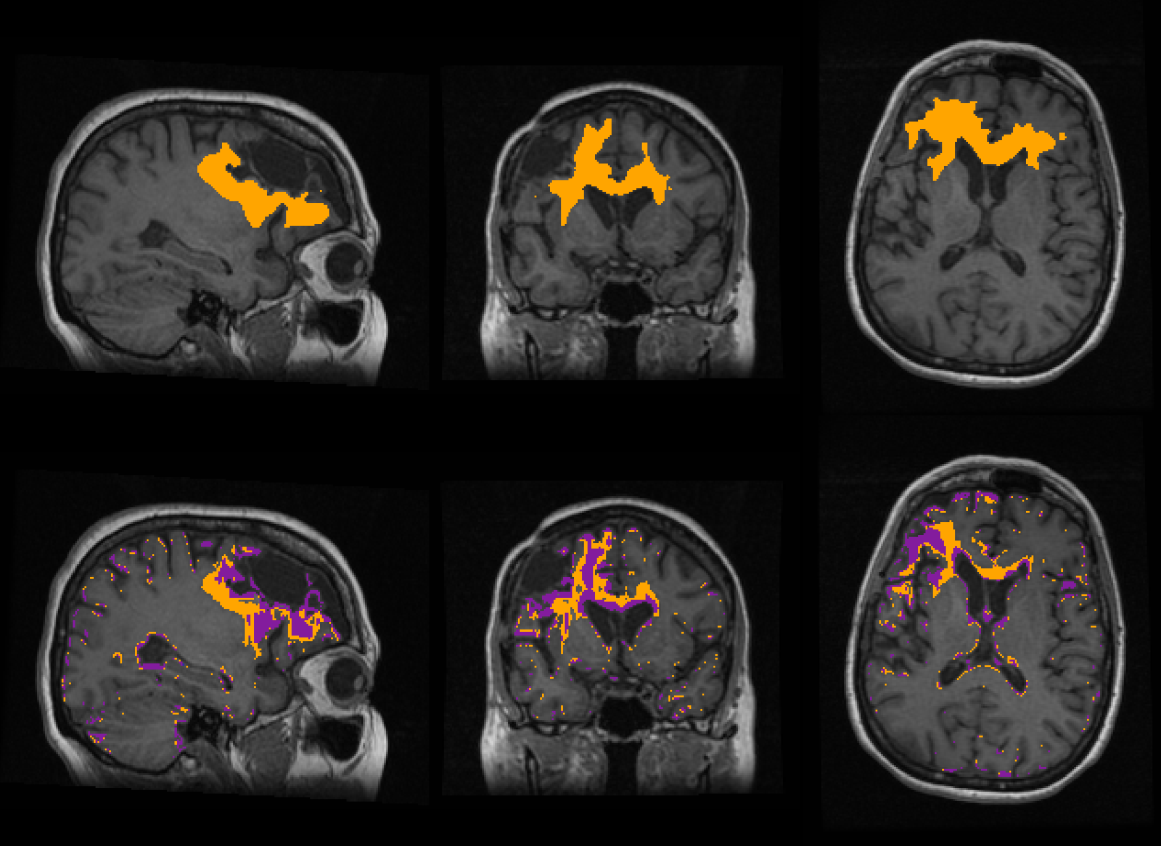


**Figure S8.** Patient 8. Sagittal, coronal and axial planes of the segmentations overlaid on the T1w scan. Top: segmentation from HD-GLIO, T2h in orange, no T1-enhancement. Bottom: ABN* in orange and T1e* in purple. The purple T1e* region is overlapped with the orange ABN*.


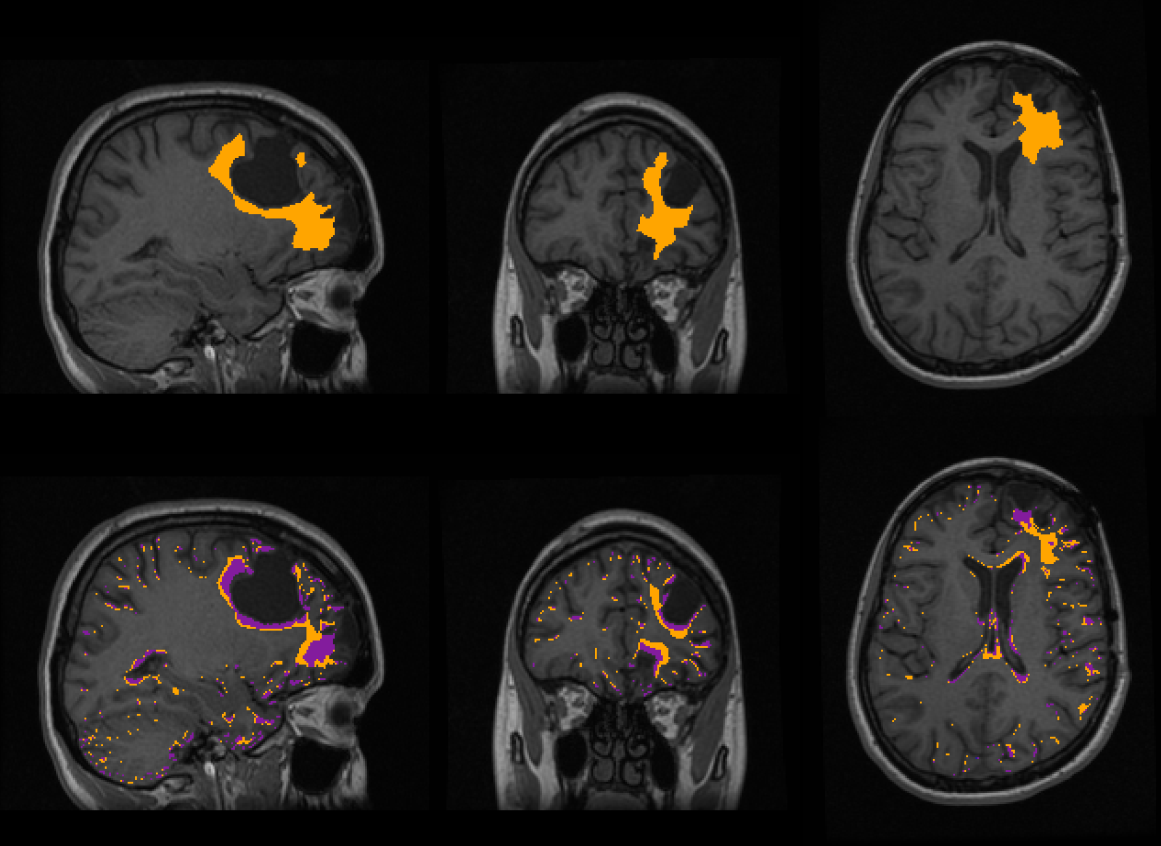


**Figure S9.** Patient 9.Sagittal, coronal and axial planes of the segmentations overlaid on the T1w scan. Top: segmentation from HD-GLIO, T2h in orange, no T1-enhancement. Bottom: ABN* in orange and T1e* in purple. The purple T1e* region is overlapped with the orange ABN*.


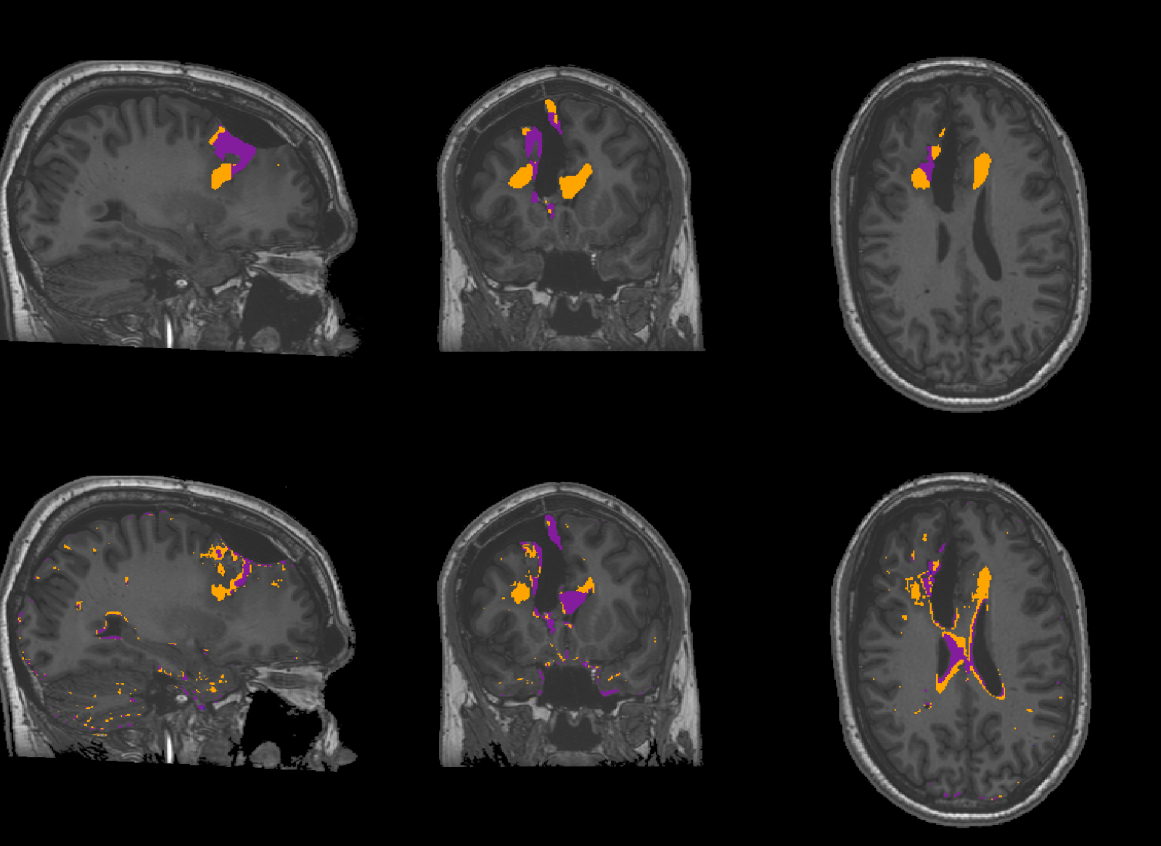


**Figure S10.** Patient 10.Sagittal, coronal and axial planes of the segmentations overlaid on the T1w scan. Top: segmentation from HD-GLIO, T2h in orange and T1e in purple. Bottom: ABN* in orange and T1e* in purple. The purple T1e* region is overlapped with the orange ABN*.


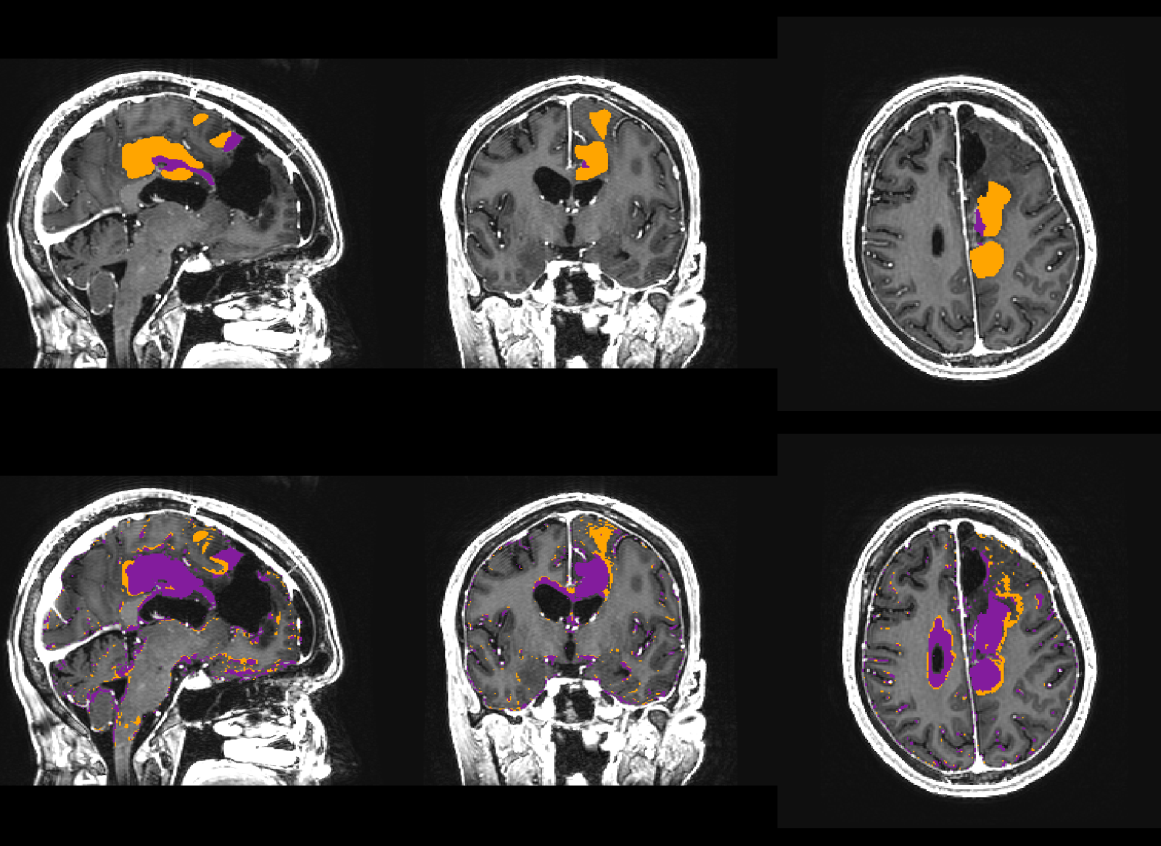


**Figure S11.** Patient 11.Sagittal, coronal and axial planes of the segmentations overlaid on the T1w scan. Top: segmentation from HD-GLIO, T2h in orange and T1e in purple. Bottom: ABN* in orange and T1e* in purple. The purple T1e* region is overlapped with the orange ABN*.


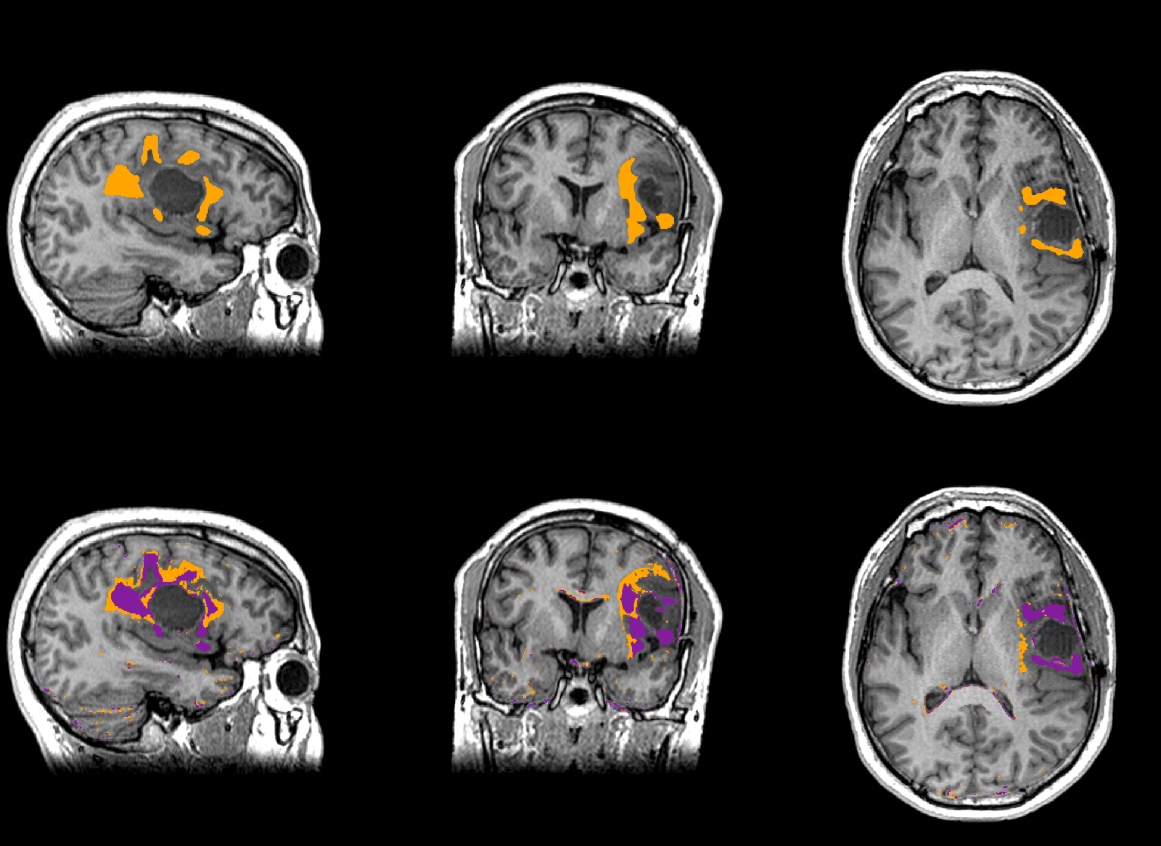


**Figure S12.** Patient 12.Sagittal, coronal and axial planes of the segmentations overlaid on the T1w scan. Top: segmentation from HD-GLIO, T2h in orange, no T1-enhancement. Bottom: ABN* in orange and T1e* in purple. The purple T1e* region is overlapped with the orange ABN*.


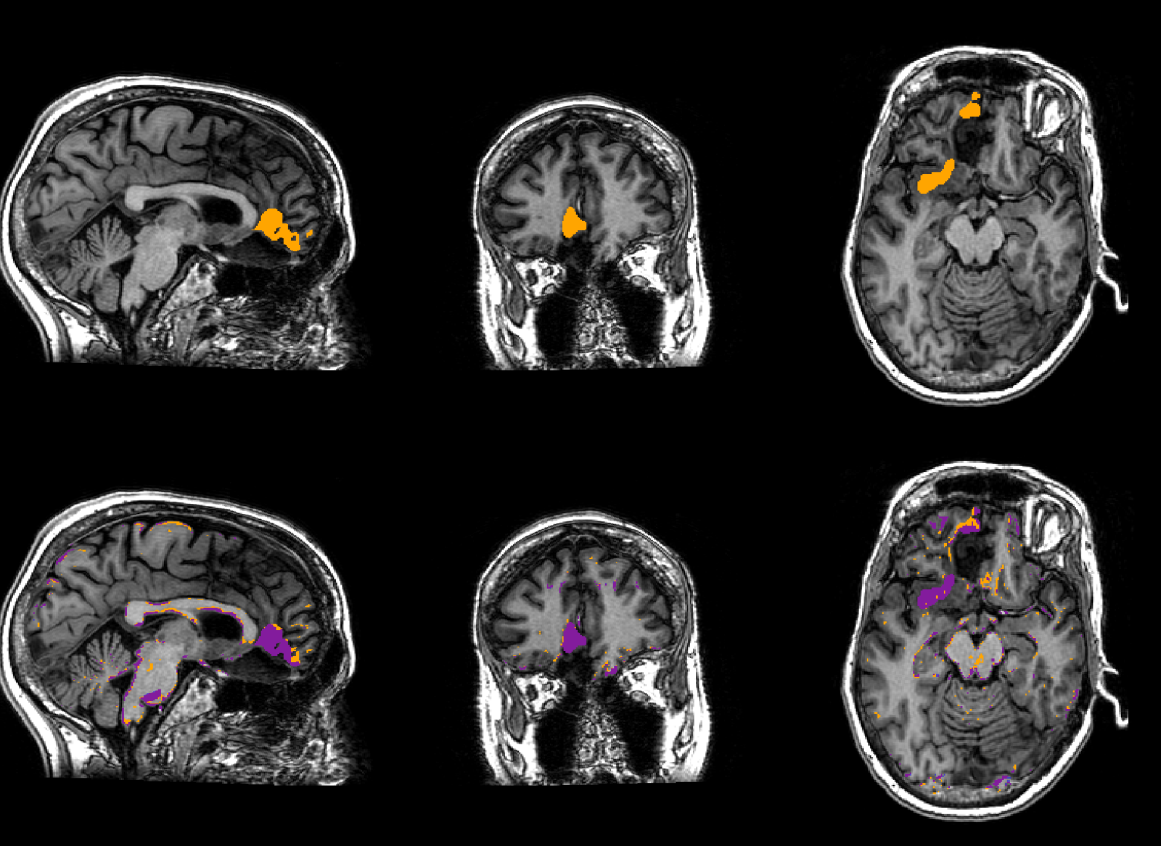


**Figure S13.** Patient 13. Sagittal, coronal and axial planes of the segmentations overlaid on the T1w scan. Top: segmentation from HD-GLIO, T2h in orange, no T1-enhancement. Bottom: ABN* in orange and T1e* in purple. The purple T1e* region is overlapped with the orange ABN*.


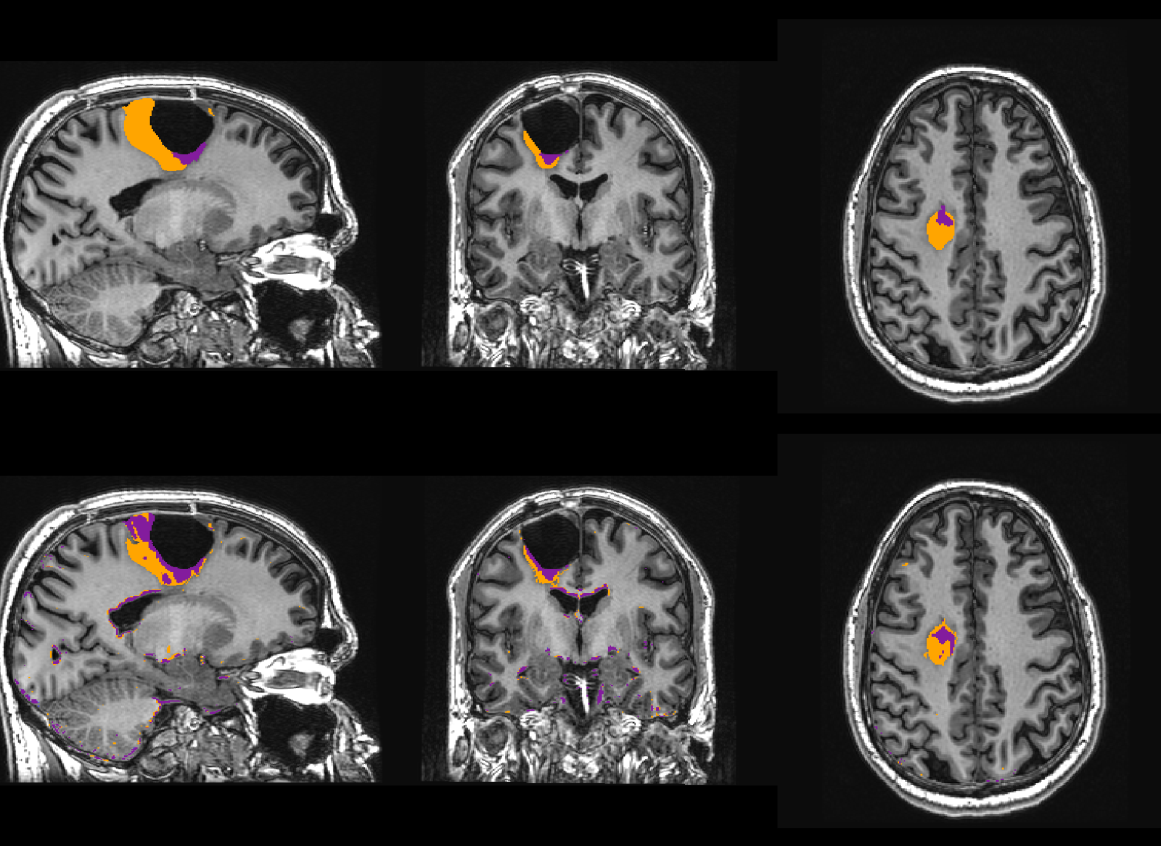


**Figure S14.** Patient 14.Sagittal, coronal and axial planes of the segmentations overlaid on the T1w scan. Top: segmentation from HD-GLIO, T2h in orange and T1e in purple. Bottom: ABN* in orange and T1e* in purple. The purple T1e* region is overlapped with the orange ABN*.
