# Supplementary material for: Pre-contrast MAGiC in treated gliomas: a pilot study of quantitative MRI
Source: Sci Rep. 2022 Dec 17;12:21820. doi: 10.1038/s41598-022-24276-5 (PMC9759533; doi:10.1038/s41598-022-24276-5)
Supplement: Supplementary file 2 — Supplementary Information 2. [file 41598_2022_24276_MOESM2_ESM.docx]

| **Patient Id** | **Age**  **(years)** | **Sex** | **Diagnosis** | **WHO**  **Grade** | **IDH** | **Treatment** | **Months since surgery** |
| --- | --- | --- | --- | --- | --- | --- | --- |
| 1 | 69 | Female | Glioblastoma | 4 | Wildtype | Chemotherapy, radiotherapy | 52 |
| 2 | 63 | Male | Astrocytoma | 3 | Wildtype | Chemotherapy, radiotherapy | 49 |
| 3 | 49 | Male | Glioblastoma | 4 | Wildtype | Chemotherapy, radiotherapy | 5 |
| 4 | 69 | Male | Glioblastoma | 4 | Mutated | Chemotherapy*, radiotherapy | 3 |
| 5 | 63 | Male | Oligodendroglioma | 2 | NOS | Radiotherapy | 122 |
| 6 | 52 | Female | Oligodendroglioma | 3 | Mutated | Chemotherapy* | 5 |
| 7 | 50 | Male | Astrocytoma | 2 | Mutated | Radiotherapy,  chemotherapy | 34 |
| 8 | 58 | Female | Oligodendroglioma | 2 | NOS | Radiotherapy,  chemotherapy* | 99 |
| 9 | 25 | Female | Glioblastoma | 4 | Wildtype | Radiotherapy,  chemotherapy | 6^S^ |
| 10 | 24 | Male | Oligodendroglioma | 2 | Mutated | Radiotherapy**,  chemotherapy** | 1 |
| 11 | 50 | Female | Astrocytoma | 2 | Mutated | Radiotherapy** | 1 |
| 12 | 37 | Male | Astrocytoma | 3 | Mutated | Radiotherapy**, chemotherapy** | 1 |
| 13 | 56 | Female | Oligodendroglioma | 2 | Mutated | Radiotherapy**,  chemotherapy** | 72 |
| 14 | 44 | Male | Astrocytoma | 2 | Mutated | Radiotherapy**,  chemotherapy** | 1 |

Supplementary Tables

**Table S1.**Patient demographics, diagnoses, WHO grade ^28,29^, Isocitrate dehydrogenase (IDH) status, treatment and months since surgery. NOS=No IDH Status available. *Treatment in progress, not finished. **Treatment planned and not applied yet. ^S^ patient 9 had the first surgery 76 months before the scan and a second surgery 39 months before the scan.

**Table S2.** Table with the statistics of the proton density (PD) values from all the patients per region of interest (ROI). The ROIs correspond to normal white matter (nWM), T2-hyperintensity (T2h), T1w-enhancement (T1e) and periabnormal area (PER). Column 3: Mean over patients of the PD per ROI statistics. Column 4: 95% confidence interval (CI) of the mean over patients of the PD per ROI statistics. Column 5: P-values of the Wilcoxon signed-rank test with the nWM (P-val ROI vs nWM). Column 6: P-values of the Wilcoxon signed-rank test with the abnormal tissue without T1w-enhancement (P-val ROI vs T2h). Column 7: P-values of the Wilcoxon signed-rank test with the T1w-enhancement voxels (P-val ROI vs T1e). * indicates p < 0.05.

| **ROI** | **ROI statistic** | **Mean (a.u.)** | **95% CI (a.u.)** | **Pval(ROI vs nWM)** | **Pval (ROI vs T2h)** | **Pval (ROI vs T1e)** |
| --- | --- | --- | --- | --- | --- | --- |
| **nWM** | Mean | 48.90 | ± 1.02 | - | < 0.01* | 0.02* |
|  | SD | 5.57 | ± 3.27 | - | < 0.01* | 0.47 |
|  | Skewness | 0.23 | ± 0.60 | - | 0.27 | 0.10 |
|  | Kurtosis | 5.57 | ± 0.36 | - | 0.02* | 1.00 |
| **T2h** | Mean | 56.20 | ± 2.98 | < 0.01* | - | 0.08 |
|  | SD | 4.00 | ± 0.52 | < 0.01* | - | 0.08 |
|  | Skewness | 0.00 | ± 0.24 | 0.27 | - | 0.03* |
|  | Kurtosis | 3.18 | ± 0.52 | 0.02* | - | 0.10 |
| **T1e** | Mean | 58.94 | ± 4.23 | 0.02* | 0.08 | - |
|  | SD | 5.08 | ± 0.62 | 0.47 | 0.08 | - |
|  | Skewness | -0.68 | ± 0.47 | 0.10 | 0.03* | - |
|  | Kurtosis | 5.15 | ± 1.48 | 1.00 | 0.10 | - |
| **PER** | Mean | 49.99 | ± 3.74 | 0.07 | < 0.01* | 0.02* |
|  | SD | 5.15 | ± 0.60 | 0.02* | 0.02* | 0.94 |
|  | Skewness | 0.33 | ± 0.30 | 0.86 | 0.04* | 0.02* |
|  | Kurtosis | 3.82 | ± 1.02 | 0.04* | 0.15 | 0.69 |

**Table S3.** Sensitivity, specificity and accuracy in % of the voxel-wise classification as T1w-enhancement (T1e*) and abnormal tissue (ABN*) for each patient in percentage.

|  | **T1w-enhancement** | | | **ABN** | | |
| --- | --- | --- | --- | --- | --- | --- |
|  | **Sensitivity** | **Specificity** | **Accuracy** | **Sensitivity** | **Specificity** | **Accuracy** |
| **Patient 1** | 65 | 78 | 78 | 73 | 68 | 69 |
| **Patient 2** | - | 89 | 89 | 55 | 84 | 82 |
| **Patient 3** | 79 | 91 | 91 | 59 | 90 | 90 |
| **Patient 4** | 90 | 91 | 91 | 49 | 86 | 85 |
| **Patient 5** | - | 89 | 89 | 61 | 86 | 85 |
| **Patient 6** | 95 | 86 | 86 | 71 | 81 | 82 |
| **Patient 7** | - | 86 | 86 | 64 | 84 | 83 |
| **Patient 8** | - | 88 | 88 | 65 | 88 | 85 |
| **Patient 9** | - | 92 | 92 | 60 | 88 | 87 |
| **Patient 10** | 59 | 96 | 96 | 62 | 94 | 93 |
| **Patient 11** | 84 | 84 | 84 | 85 | 80 | 81 |
| **Patient 12** | - | 93 | 93 | 99 | 91 | 91 |
| **Patient 13** | - | 95 | 95 | 99 | 93 | 93 |
| **Patient 14** | 75 | 95 | 95 | 85 | 95 | 94 |
| **Mean (95% CI)** | 77 (±7.5) | 89 (±2.8) | 89 (±2.8) | 69 (±9.1) | 86 (±4.0) | 85 (±3.8) |

**Table S4.** First row indicates if the abnormal tissue showed T1w-enhancement (Y) or not (N). The following rows report the number of times that the patient was classified as having T1w-enhancement during the leave-pair-out cross-validation, which makes 7 predictions per patient. Patients with T1w-enhancement are marked using columns in green. Numbers in red indicates that the enhancement was misclassified the majority of the times. The validation was done using only the T1 values, only the T2 values, the Euclidian norm of the T1 and T2 values (normT1T2) and the Euclidian norm of the logarithm of the T1 and T2 values (normlog).

|  | **Param** | **Pat 1** | **Pat 2** | **Pat 3** | **Pat 4** | **Pat 5** | **Pat 6** | **Pat 7** | **Pat 8** | **Pat 9** | **Pat 10** | **Pat 11** | **Pat 12** | **Pat 13** | **Pat 14** |
| --- | --- | --- | --- | --- | --- | --- | --- | --- | --- | --- | --- | --- | --- | --- | --- |
| **T1-enhan** | - | Y | N | Y | Y | N | Y | N | N | N | Y | Y | N | N | Y |
| **T1** | Mean | 0 | 0 | 7 | 0 | 0 | 7 | 0 | 0 | 0 | 7 | 7 | 7 | 7 | 1 |
|  | SD | 0 | 0 | 0 | 7 | 7 | 0 | 0 | 0 | 0 | 7 | 1 | 0 | 0 | 7 |
|  | Skewness | 7 | 7 | 7 | 0 | 0 | 1 | 6 | 0 | 0 | 7 | 0 | 0 | 0 | 7 |
|  | Kurtosis | 7 | 7 | 1 | 0 | 0 | 7 | 6 | 0 | 0 | 7 | 7 | 0 | 0 | 7 |
| **T2** | Mean | 0 | 0 | 0 | 0 | 6 | 3 | 0 | 4 | 4 | 5 | 7 | 7 | 7 | 5 |
|  | SD | 0 | 0 | 0 | 0 | 0 | 0 | 0 | 0 | 0 | 7 | 0 | 0 | 0 | 6 |
|  | Skewness | 7 | 7 | 7 | 0 | 0 | 3 | 7 | 7 | 7 | 7 | 3 | 0 | 0 | 3 |
|  | Kurtosis | 7 | 7 | 7 | 0 | 5 | 6 | 7 | 7 | 6 | 6 | 6 | 0 | 0 | 0 |
| **normT1T2** | Mean | 0 | 0 | 7 | 0 | 0 | 7 | 0 | 0 | 0 | 7 | 7 | 7 | 7 | 1 |
|  | SD | 0 | 0 | 0 | 7 | 7 | 0 | 0 | 0 | 0 | 7 | 1 | 0 | 0 | 7 |
|  | Skewness | 7 | 7 | 7 | 0 | 0 | 2 | 6 | 0 | 5 | 7 | 0 | 0 | 0 | 7 |
|  | Kurtosis | 7 | 7 | 1 | 0 | 0 | 7 | 6 | 0 | 0 | 7 | 7 | 0 | 0 | 7 |
| **normlog** | Mean | 0 | 0 | 7 | 0 | 0 | 7 | 0 | 0 | 0 | 7 | 7 | 7 | 7 | 7 |
|  | SD | 0 | 0 | 7 | 7 | 7 | 1 | 7 | 7 | 7 | 7 | 7 | 0 | 6 | 7 |
|  | Skewness | 7 | 7 | 7 | 0 | 0 | 0 | 0 | 0 | 0 | 7 | 0 | 0 | 0 | 7 |
|  | Kurtosis | 7 | 7 | 7 | 0 | 0 | 2 | 6 | 0 | 5 | 7 | 0 | 0 | 0 | 7 |
